# *Pulsatilla chinensis* Saponins Ameliorate Inflammation and DSS-Induced Ulcerative Colitis in Rats by Regulating the Composition and Diversity of Intestinal Flora

**DOI:** 10.3389/fcimb.2021.728929

**Published:** 2021-11-05

**Authors:** Yali Liu, Mingyue Zhou, Ming Yang, Chen Jin, Yonggui Song, Jingbin Chen, Meng Gao, Zhifu Ai, Dan Su

**Affiliations:** ^1^ Key Laboratory of Depression Animal Model Based on Traditional Chinese Medicine (TCM) Syndrome, Jiangxi Administration of Traditional Chinese Medicine, Key Laboratory of TCM for Prevention and Treatment of Brain Diseases with Cognitive Impairment, Jiangxi University of Chinese Medicine, Nanchang, China; ^2^ Department of Pharmacy, Nanchang Medical College, Nanchang, China; ^3^ Key Laboratory of Modern Preparation of Traditional Chinese Medicine, Ministry of Education, State Key Lab of Innovation Drug and Efficient Energy-Saving Pharmaceutical Equipment, Jiangxi University of Traditional Chinese Medicine, Nanchang, China

**Keywords:** *Pulsatilla chinensis* saponins (PRS), 16S rRNA, gut microbiome, ulcerative colitis (UC), dextran sulfate sodium (DSS)

## Abstract

*Pulsatilla chinensis* (Bunge) Regel is a commonly used Chinese medicine for clearing away heat and detoxification, cooling blood, stopping dysentery, and anti-inflammatory effects. *Pulsatilla chinensis* saponins (PRS) have been identified to be responsible for producing these pharmacological activities. Studies have shown that Pulsatilla decoction has a good therapeutic effect on ulcerative colitis (UC), however, the therapeutic effect of PRS on UC has not been reported. Therefore, the purpose of this study was to investigate the possible anti-UC activity of PRS using a dextran sulfate sodium (DSS)-induced rat model, and further study the mechanism of PRS in the treatment of UC. The fecal and colon samples were collected from rats to monitor the changes in the composition and diversity of the intestinal flora, and pathological colon sections were also made to examine the mesenteric hemorheological characteristics. The results showed that PRS significantly reduced the mesenteric blood flow in UC rats and significantly alleviated the inflammatory response, which indicates that saponins are involved in the anti-UC effects of PRS. At the same time, it is also suggested that the regulation of intestinal flora by *Pulsatilla chinensis* saponins is an important pathway for its anti-UC activity, which may be ascribed to the increase in beneficial bacteria like norank_F_Muribaculaceae and norank_F_norank_O_Clostridia_UCG-014, and decrease in the harmful Bacteroides.

## Introduction

*Pulsatilla chinensis* (Bunge) Regel, an antipyretic traditional Chinese medicine (TCM), has a rich history dating back centuries for medicinal practices in China and some oriental countries (State Pharmacopoeia Commission, 2020). It has been officially listed in the Chinese Pharmacopoeia for a long time and has the effect of clearing away heat and toxin, cooling blood, and stopping dysentery (Xu et al., 2011). *Pulsatilla chinensis* (*P.*chinensis) is widely used to treat schistosomiasis, inflammatory bacterial infections (Liu et al., 2014; Tong et al., 2017). Currently, it has gained extensive popularity in the Chinese medicine system due to its excellent anti-inflammatory properties. *Pulsatilla* Decoction has been widely reported being used for the treatment of UC (Guisen, 2015; Wang et al., 2016). Triterpenoid saponins are the main medicinal components of *Pulsatilla chinensis* (Bunge) Regel (Song et al., 2021). *Pulsatilla* triterpenoid saponins have many pharmacological activities, including anti-tumor, anti-inflammatory, anti-oxidation, anti-virus, anti-schistosome, immune enhancement, and other pharmacological activities (Su et al., 2020). However, the therapeutic effect of PRS on UC has not been reported (Wang et al., 2016).

UC is a chronic and repeatedly occurring colorectal inflammatory disease (Ungaro et al., 2016). Although the specific etiology is still unclear, but immune dysfunction, individual genetic diversity, intestinal flora disorders, and other factors are closely linked to the pathogenesis of UC (Shen et al., 2018). In recent years, gut flora dysbiosis has received increasing attention for inducing UC (Wirtz and Neurath, 2017). The intestinal microflora is characterized by a higher species diversity (Feng et al., 2019), which gradually colonizes the gastrointestinal tract from the moment an individual is born and stays with an individual for a whole life. Because of its significant involvement in the human body’s physiological functions and pathologies, the intestinal flora is, to some extent, the largest “organ” in the body (Jiang et al., 2018).

Recently, it has been believed the intestinal flora is involved in the occurrence and development of UC because the imbalance of intestinal flora (beneficial and harmful bacteria) leads to the increase in intrinsic epithelial permeability, which results in the occurrence of intestinal inflammatory diseases (Oshitani et al., 2003; Huang et al., 2017). The number of bacteria present in the colon is significantly higher than in other parts of the gastrointestinal tract, with a concentration of about 10^9^—10^12^ CFU/mL, mainly including anaerobic bacteria, Bacteroides, and other dominant groups (Borody et al., 2003). They maintain a symbiotic or antagonistic relationship and are closely related to the host health and disease state. For example, if abnormal changes occur in the species and quantity of normal intestinal flora will result in an imbalance of intestinal flora, leading to UC (Venturi et al., 1999). In recent years, studies have found that saponins have a good regulatory effect on the flora (Frank and Pace, 2008; Himmel et al., 2012; Shan et al., 2020). Therefore, we speculate that it may be of great significance to explore the pharmacological effect of PRS on UC. This study was aimed to explore the anti-inflammatory efficacy of PRS on DSS-induced UC, to understand its mechanism by monitoring the changes in the composition and diversity of intestinal flora in rats.

## Materials and Methods

### Animals

Animal care was as per the Guidelines for Animal Experiments of Jiangxi University of Chinese Medicine (Nanchang), and the experimental protocol was approved by the University’s Animal Ethics Committee. Male Sprague-Dawley rats (n=6, 180-220 g) were housed in a sterile environment and were fed autoclaved chow and clean water with a 12 h light/12 h dark cycle, constant temperature (21°C-22°C), and controlled humidity (55 ± 5%).

### Reagents and Instruments

Dried Radix of *P*. chinensis was purchased from a Chinese herbal medicine store in Suzhou and identified by Prof. Xiaoran Li of the School of pharmacy of Suzhou University. The voucher specimen (No. 08-02-15-18) has been deposited at Soochow University. The other materials used in the study were bought from different companies as follows; Salicylazosulfapyridine (SASP) from (Shanghai Xinyi Tianping Pharmaceutical Co., Ltd., gyzz H31020557); CMC-Na (Carboxymethylcellulose sodium) (Xilong Chemical Co., Ltd.), Dextran sulfate sodium (DSS) from (MP Biomedical, LLC); chloral hydrate, and paraformaldehyde from (Shanghai Aladdin Biochemical Technology Co., Ltd.); KQ-250DB numerical control ultrasonic cleaner from (Gongyi Yuhua Instrument Co., Ltd.); Milli-Q ultrapure water machine from (Millipore company); 4°C refrigerator from (Haier company); –80°C ultra-low temperature refrigerator from (Thermo Fisher company); KDM type temperature control electric heating set from (juancheng Hualu electric heating instrument Co., Ltd.); SY-2000 rotary evaporator from (Shanghai Yarong biochemical instrument factory); SHB-III circulating water multi-purpose vacuum pump from (Zhengzhou Great Wall Technology Industry and Trade Co., Ltd.); and Doppler infrared imager from (Model: MOORLDI2-HIR, Moor Instruments Ltd Millwey Rise, AXMINSTER DEVON, EX13 5HU UK).

### Preparation of PRS Extract

The dried and crushed raw materials of the *Pulsatilla chinensis* (Bunge) Regel were processed as follows: 2500 g of *P*. chinensis was extracted with 70% ethanol for 3 times successively, macerated for 4 h, and then dried under reduced pressure conditions. The 280 g residue was chromatographically separated using a D101 resin column with water-ethanol gradient elution. The fractions eluted using 60% ethanol were lyophilized to obtain a powder (PRS, 125 g). The ethanolic extracts of PRS were redissolved in an aqueous solution with 0.5% sodium carboxymethylcellulose (CMC-Na) to obtain a uniformly dispersed suspension of 18.75 mg/mL for oral dosage (Liu et al., 2013; Song et al., 2019).

### Grouping and Establishment of UC Rat Model

SD rats were randomly divided into 6 groups after adaptive feeding for 7 days, and each group had 6 animals: control group, model group, PRSH (*Pulsatilla chinensis* saponins high dose group) (400 mg/kg), PRSM (*Pulsatilla chinensis* saponins middle dose group) (300 mg/kg), PRSL (*Pulsatilla chinensis* saponins low dose group) (200 mg/kg), and positive control SASP group (400 mg/kg) (Wang et al., 2020).

The UC treatment methods were as follows: the rats in the model group drank 4% DSS freely and were given 0.5% CMC-Na by gavage every day; the rats in the control group drank freely and were given 0.5% CMC-Na by gavage every day, and the rats in the SASP group drank 4% DSS freely. The three PRS dose groups of rats were free to drank 4% DSS, and according to the actual consumption, 4% DSS was freshly constituted every day during the experiments. The rats in each group were weighed regularly every day, and the stool characteristics, occult blood or bloody stool, hair, activity, and deaths were closely observed. The rats were fasted for 12 hours before sacrificing on the 9th day of treatment.

### Sample Collection and Macroscopic Score of Colonic Tissues

The fresh faecal samples of rats were collected on an aseptic operation platform before sacrificing the animals on the 9th day of the treatment. Colonic tissues were taken out immediately after sacrificing the animals, and the length and pathological changes were recorded and then washed with phosphate buffered saline (PBS) buffer solution, dried with filter paper, and fixed in 10% paraformaldehyde. The freshly collected feces and fixed colonic tissues were stored in the refrigerator at –80°C until further analysis. For tissue sectioning, the colonic tissue was processed by following steps of repair, washing, dehydration, transparency, wax soaking, embedding, sectioning, staining, and transparency, and sealing to make the tissue section (4 μm thickness) with routine hematoxylin staining (HE) staining, and then the histological changes were observed using a light microscope.

### HE Staining to Detect the Pathological Changes of Colon

The colonic tissues were fixed in 4% paraformaldehyde for more than 24 hours before analysis. After being dehydrated with gradient ethanol and cleared with xylene, the tissues were embedded in wax, sectioned (thickness 4 μm), followed by dewaxing, hematoxylin staining for 5 min, PBS washing, differentiation in 1% hydrochloric acid ethanol, eosin staining for 30 s, gradient ethanol dehydration, transparent treatment, neutral glue sealing, and then finally examined under a microscope to record the pathological changes in the tissues.

### Processing of 16S rRNA Gene Sequences

The Illumina MiSeq platform was paired-sequenced equimolar and depurated amplicons (2×300). Demultiplexing original fastq files, quality-filtering in Trimmomatic and combining on FLASH, and following regulations like: (i) Over a 50 bp sliding window were cut off the reads at any position where medial quality scores are more than 20 bp. (ii) Primers were matched exactly with two nucleotides mismatching and removing ambiguous bases in reads. (iii) Overlapping sequences, more than 10 bp, were combined based on their own sequences by UPARSE (version 7.1 http://drive5.com/uparse/) and were grouped with 97% similarity cutoff Operational taxonomic units (OTUs) and by UCHIME were separated and undocked chimeric sequences. Moreover, analyzed by the RDP Classifier algorithm (http://rdp.cme.msu.edu/), each 16S rRNA gene sequence taxonomy which was used to build digests of the taxonomic distributions of OTUs to compute relative abundances of microbiota at several diverse ranks was against the Silva (128) 16S rRNA database and with a confidence threshold of 70%. These taxonomies were used to construct summaries of the taxonomic distributions of OTUs, which can then be applied to calculate microbiota’s relative abundances at different levels. Distance matrices (Beta diversity) between samples were generated on the basis of weighted (Bray-Curtis similarity) reported according to principal coordinate analysis (PCoA).

### Statistical Methods

The data were analyzed with GraphPad Prism 8 statistical software package, and the data were expressed as mean ± SEM. The data between the two groups were compared by student’s t-test, and one-way ANOVA was employed to analyze the differences between the two groups. The intestinal microflora data were statistically analyzed using the bio cloud platform of Shanghai Meiji Biomedical Technology Co., Ltd. And P < 0.05 indicates that the differences are statistically significant.

## Results

### Effects of PRS on the Morphology of UC Rats

#### Changes in Behavior and Body Weight in Rats

**Figure 1:**
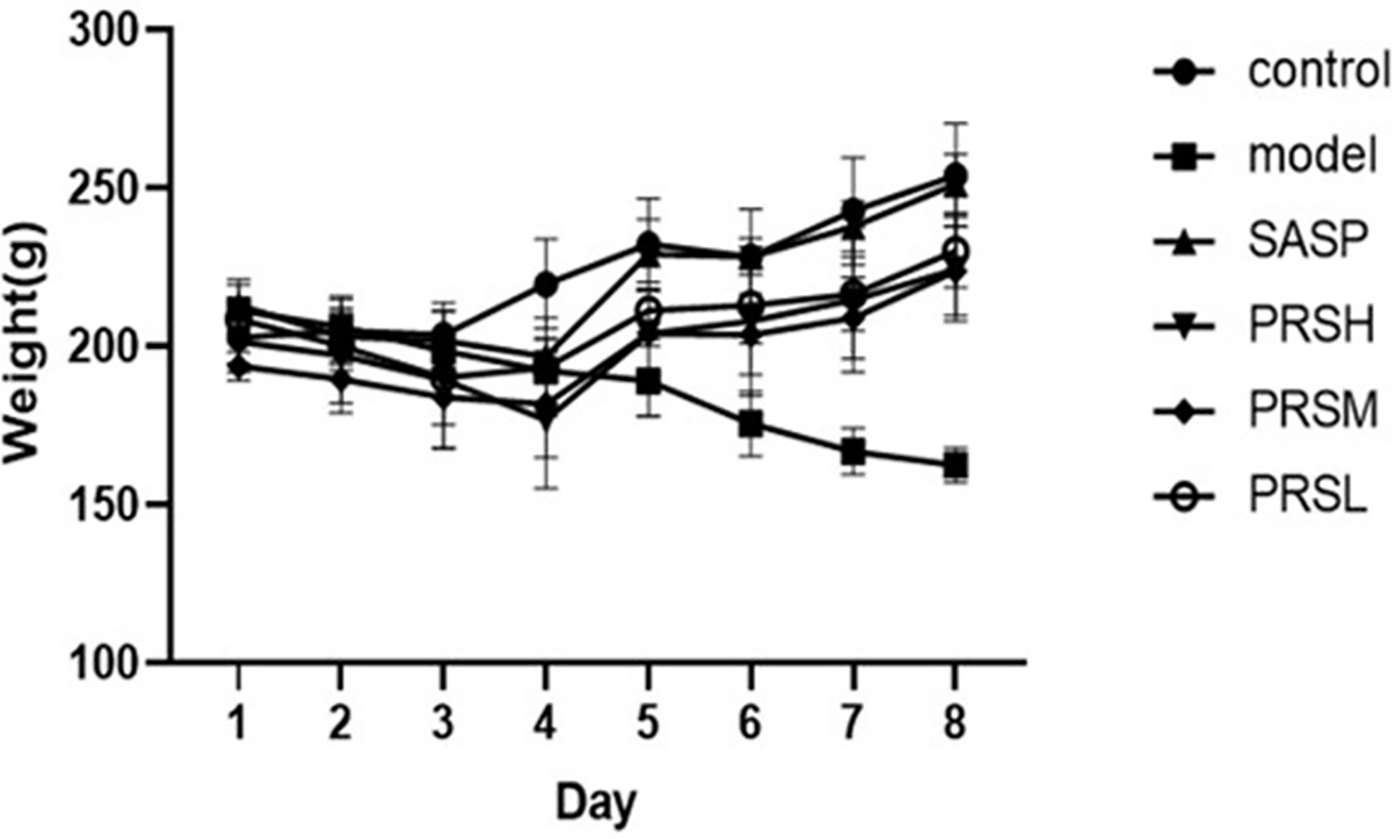
Change of body weight of mice from different groups during modeling period.

The changes in the behavior and weight of rats were regularly monitored throughout the experimental study duration. The rats in the control group had bright hair color, a normal diet, a good mental state, and no bloody stool. On the sixth day, the rats in the model group, SASP group, and PRS groups appeared hematochezia successively; however, no rats had died during the administration. The model group’s body weight showed a downward trend, and the control group showed an upward trend. In the first four days, the SASP group’s bodyweight and PRSH, PRSM, and PRSL groups showed a downward trend and increased from the fourth day. And the rats were sacrificed on the 9th day, and the results are shown in **Figure 1**.

### Effect of PRS on Pathological Tissue of UC Rats

**Figure 2:**
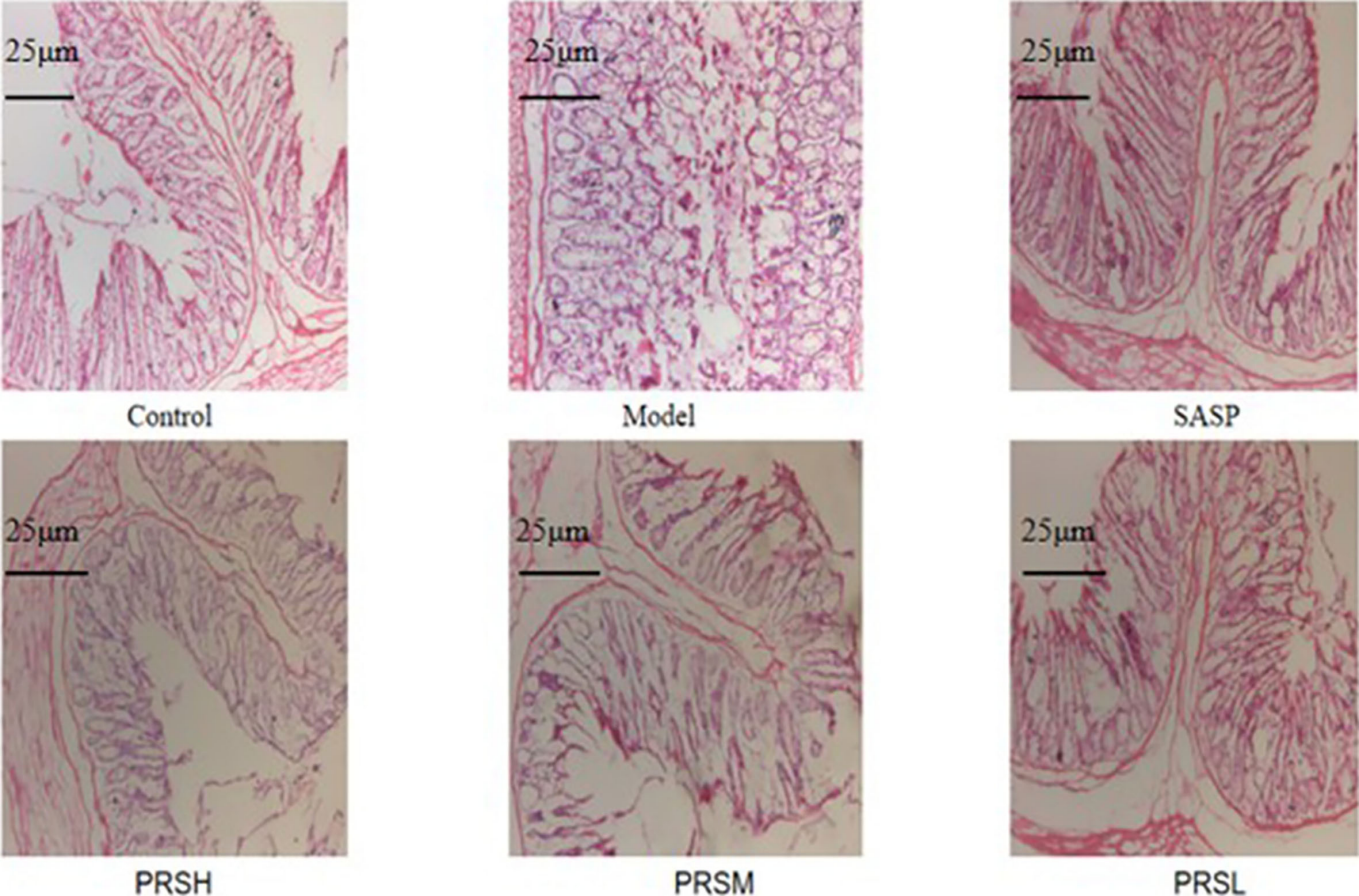
Histopathological changes were improved by PRS saponins. The colons of each group were processed for histological evaluation (H&E staining 100×). The scale bar is 25 μm.

**Figure 3:**
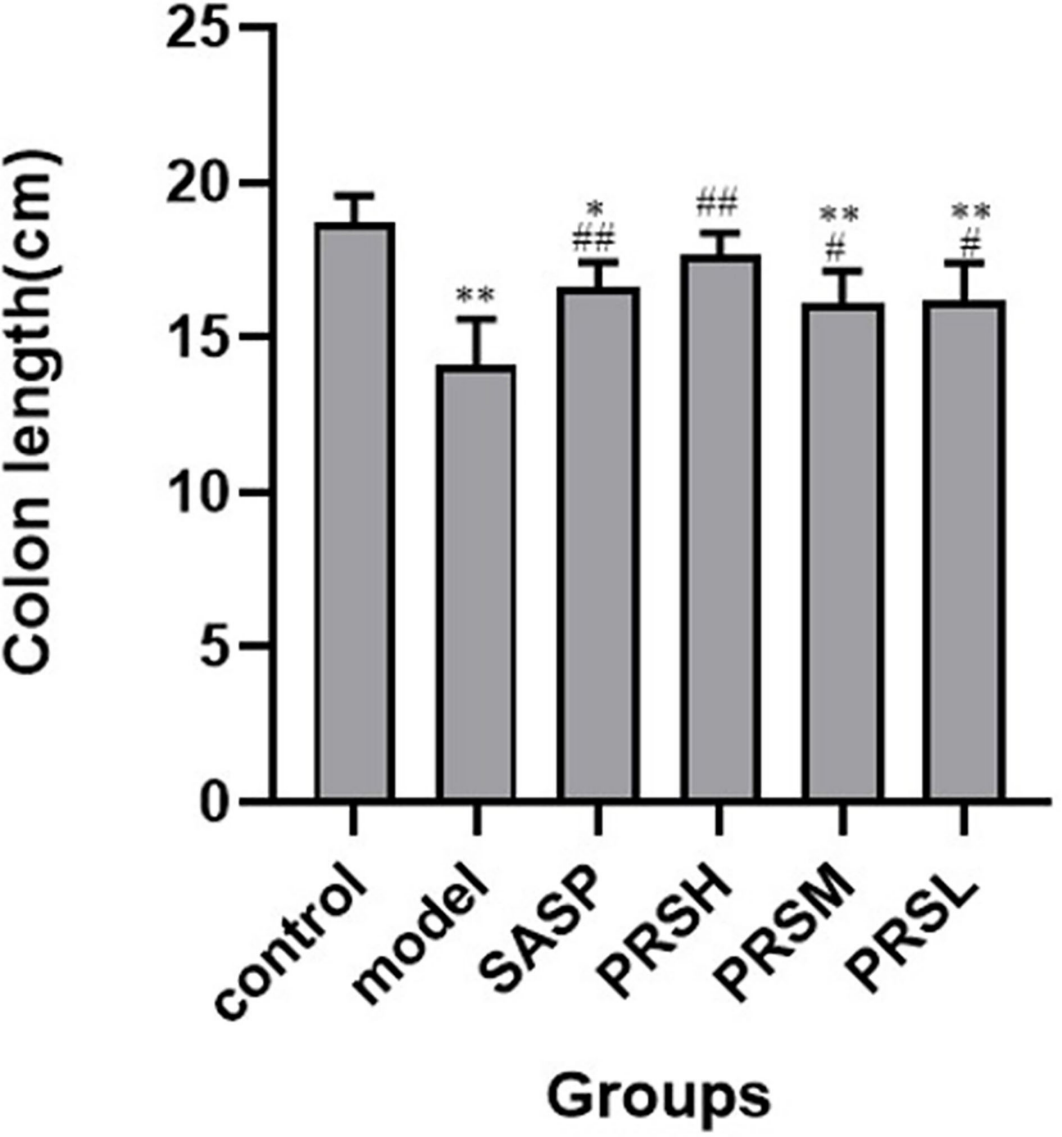
Lengths of colons from each group of rat. Data are presented as means ± SD (n = 6 per group). ^#^p < 0.05 and ^##^p < 0.01 vs the model group on the same day; *p < 0.05 and **p < 0.01 vs the control group.

**Figure 4:**
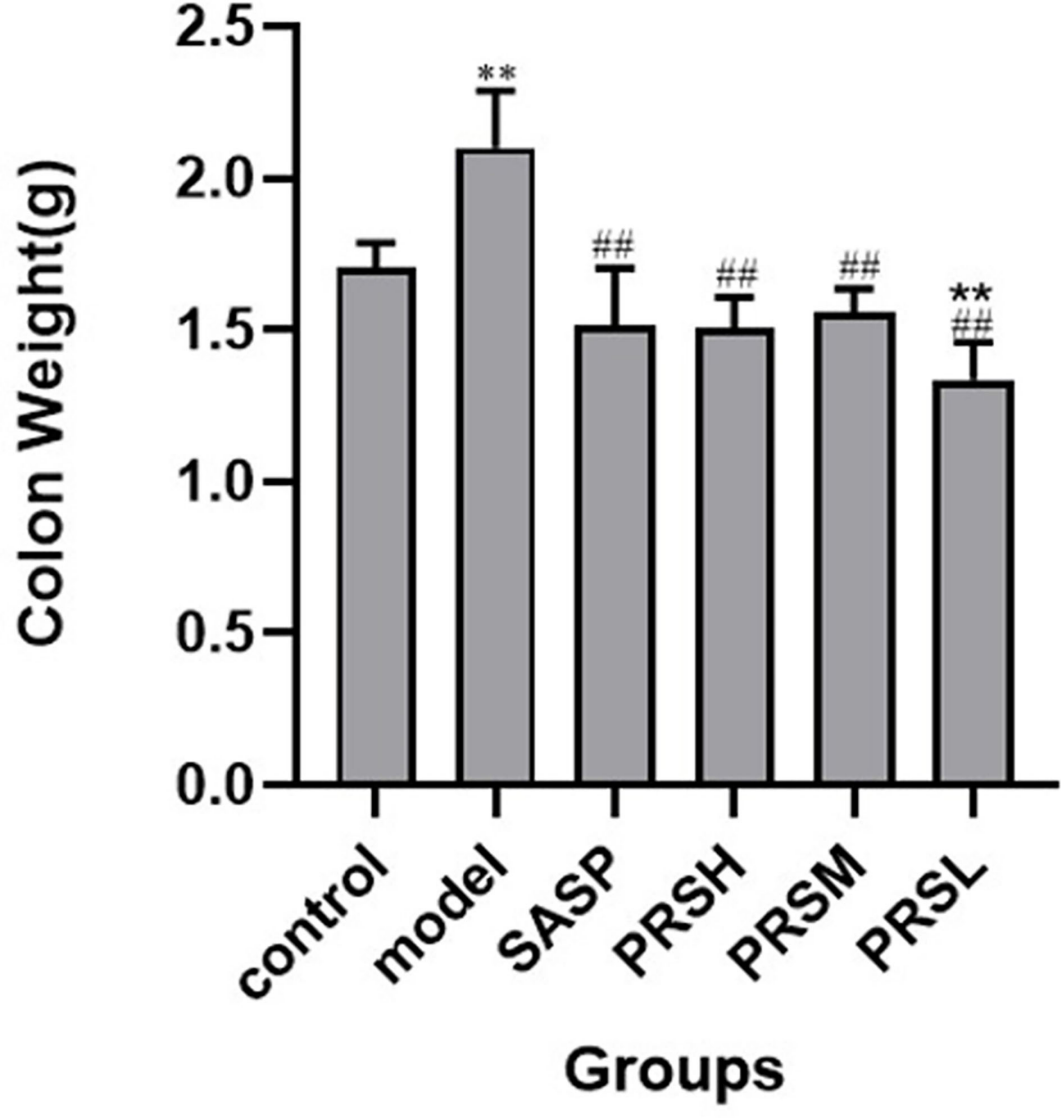
Weight of colons from each group of rat. Data are presented as means ± SD (n= 6 per group). ^#^p < 0.05 and ^##^p < 0.01 vs the model group on the same day; *p < 0.05 and **p < 0.01 vs the control group.

The effect of PRS on the DSS-induced UC rat model was monitored by noticing the changes in the mucosa and other changes in the surrounding cells of the colonic tissues. In the control group, the structure of colonic mucosa was normal, the large intestine glands were closely arranged, the epithelial cells were normal without ulcer, and there were more goblet cells. In the model group, ulcers were found in the superficial layer of colonic mucosa, and the tissue structure of the mucosal layer disappeared, and inflammatory cell infiltration was observed. In the SASP group, the colonic mucosa structure was improved, no obvious ulcer was found, no obvious injury of epithelial cells, and no obvious infiltration of inflammatory cells. Similarly, the structure of colonic mucosa was improved in the high-dose PRSH group, and no obvious ulcer was found. In the PRSM group, the structure of colonic mucosa was also improved, with no obvious ulcers, and the epithelial cells were not significantly damaged, and goblet cells were reduced. In the low-dose PRSL group, the structure of colonic mucosa was improved, no obvious ulcer was found, but the local glandular epithelial cells were damaged, and goblet cells were reduced, and the results are shown in **Figure 2**. Furthermore, there was no significant difference in colon length between the high-dose PRSH group and the control group, but there were significant differences between the other groups and the control group; there were significant differences in colonic weight between the low-dose PRSL group and the model group, but there was no significant difference between the other groups and the control group. The results are shown in **Figures 3**, **4**.

### Effect of PRS on Mesenteric Blood Flux in UC Rats

**Figure 5:**
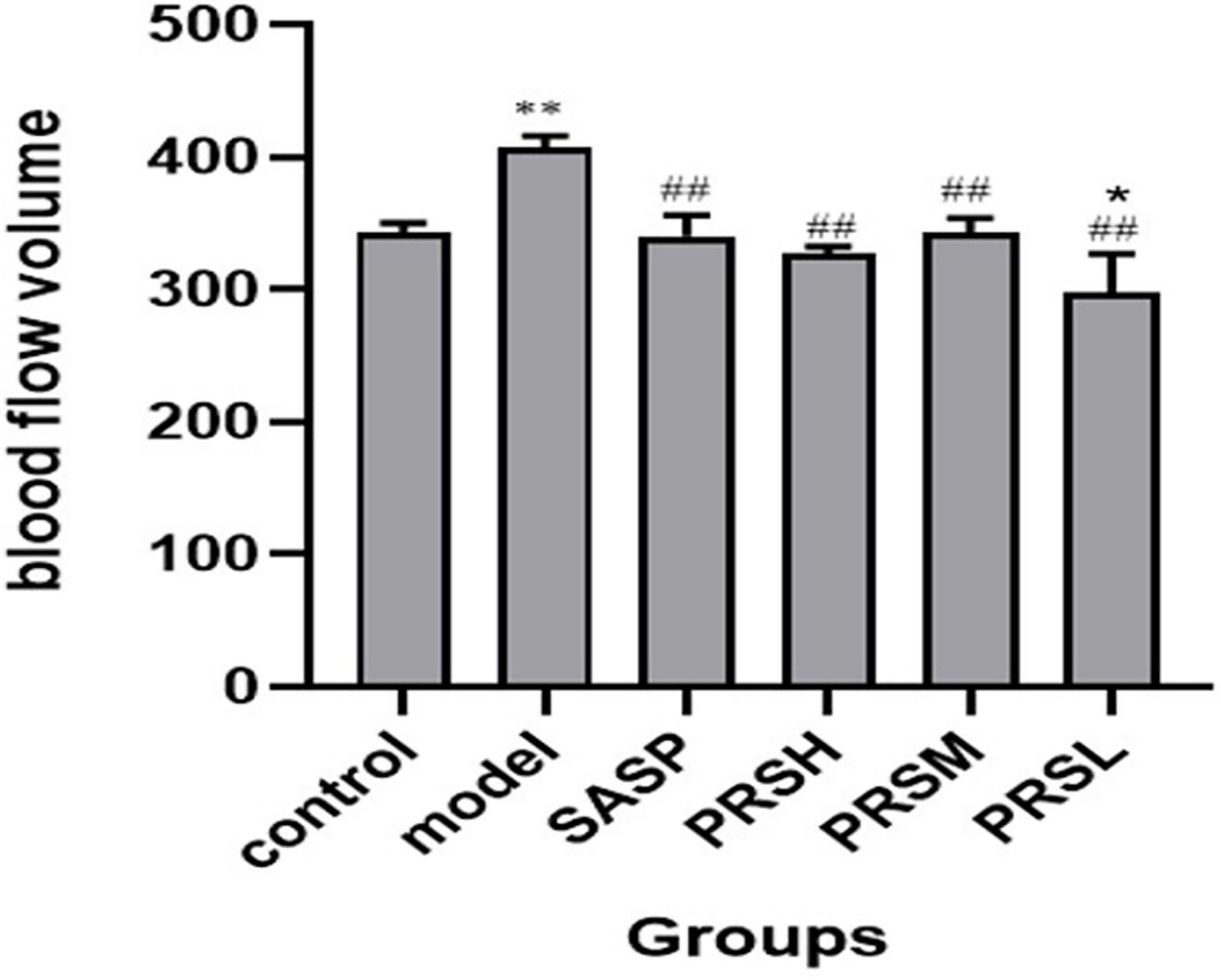
Blood flow volume of each group of rat. Data are presented as means ± SD (n= 6 per group). ^##^p < 0.01 vs the model group on the same day; *p < 0.05 and **p < 0.01 vs the control group.

**Figure 6:**
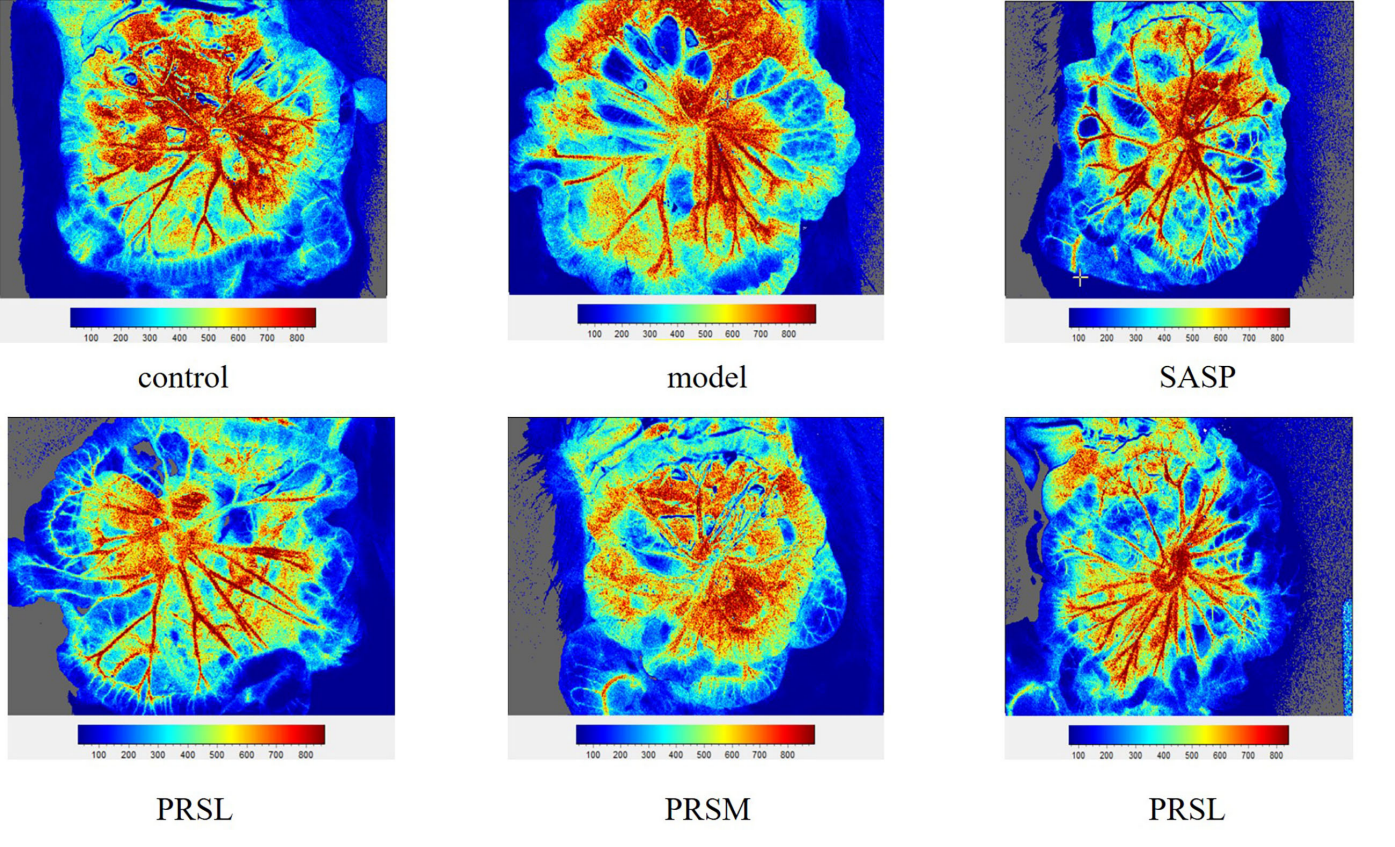
Mesenteric blood flow volume of each group of rat. Data are presented as means ± SD (n= 6 per group).

UC is a chronic infectious gastrointestinal disease, and severely affected patients can develop hypokalemia, shock, intestinal perforation, and the clinical manifestations are both active and inactive. It has been reported that the severity of UC can be reflected by observing the mesentery. Therefore, the effect of PRS was also studied by examining the changes in the mesentery, and the results of hemorheology showed that compared with the control group, the mesenteric blood flow in the model group was significantly increased, the mesenteric blood flow in the PRSL group was significantly decreased, and there was no significant difference in the mesenteric blood flow among SASP group, PRSH group, and PRSM group. Compared with the model group, the other five groups’ mesenteric blood flow decreased significantly (Bloemendaal et al., 2020). The hemorheology results showed that both PRSH and PRSM groups could significantly reduce mesenteric blood flow in rats with DSS-induced UC. It can be seen that PRS can improve the mesenteric ulcer induced by DSS and reduce the inflammatory reaction in UC rats, as shown in **Figures 5**, **6**.

### Effect of PRS on Intestinal Flora of UC Rats

**Figure 7:**
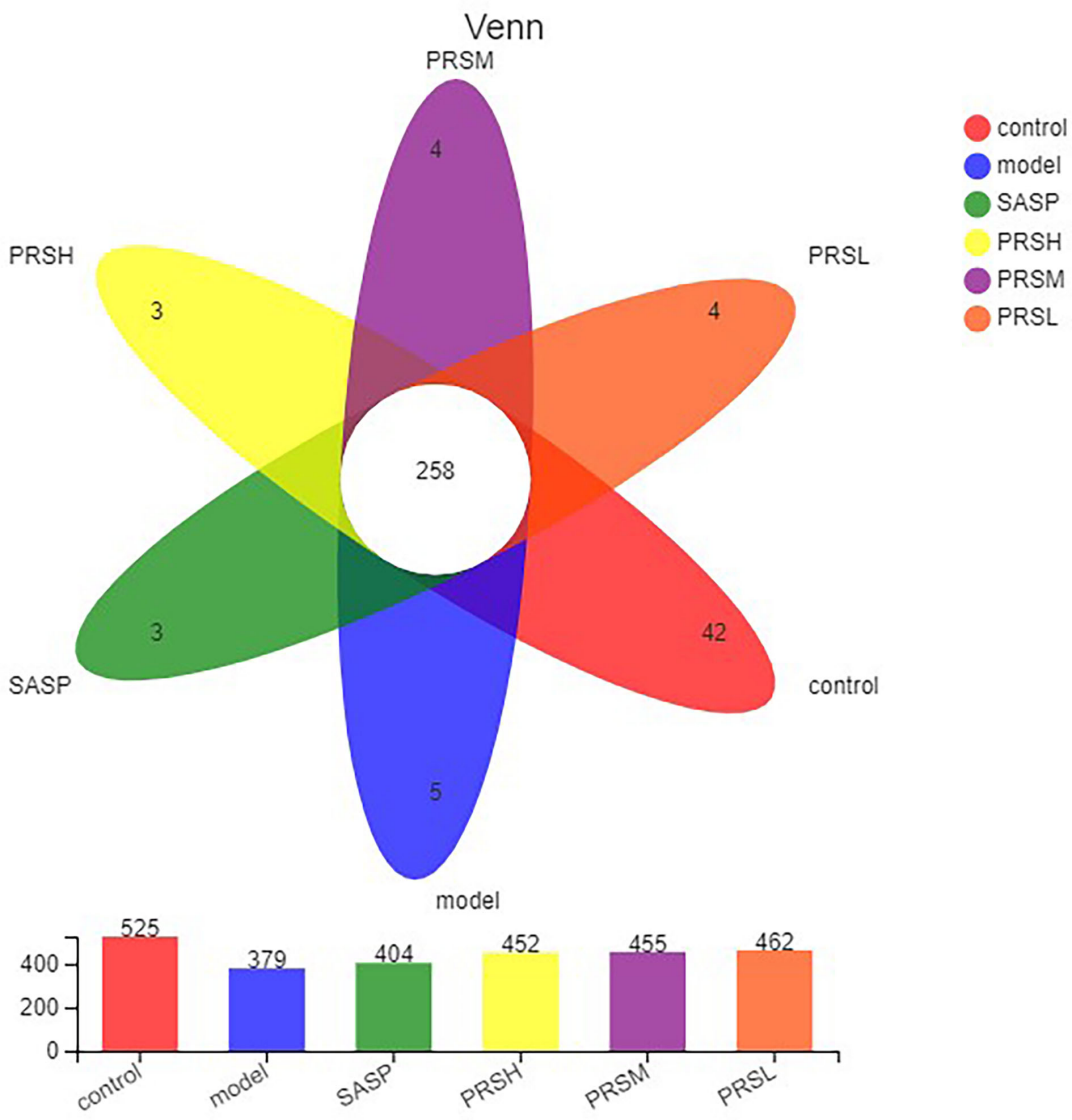
OTU analysis of intestinal microbes in UC rats.

**Figure 8:**
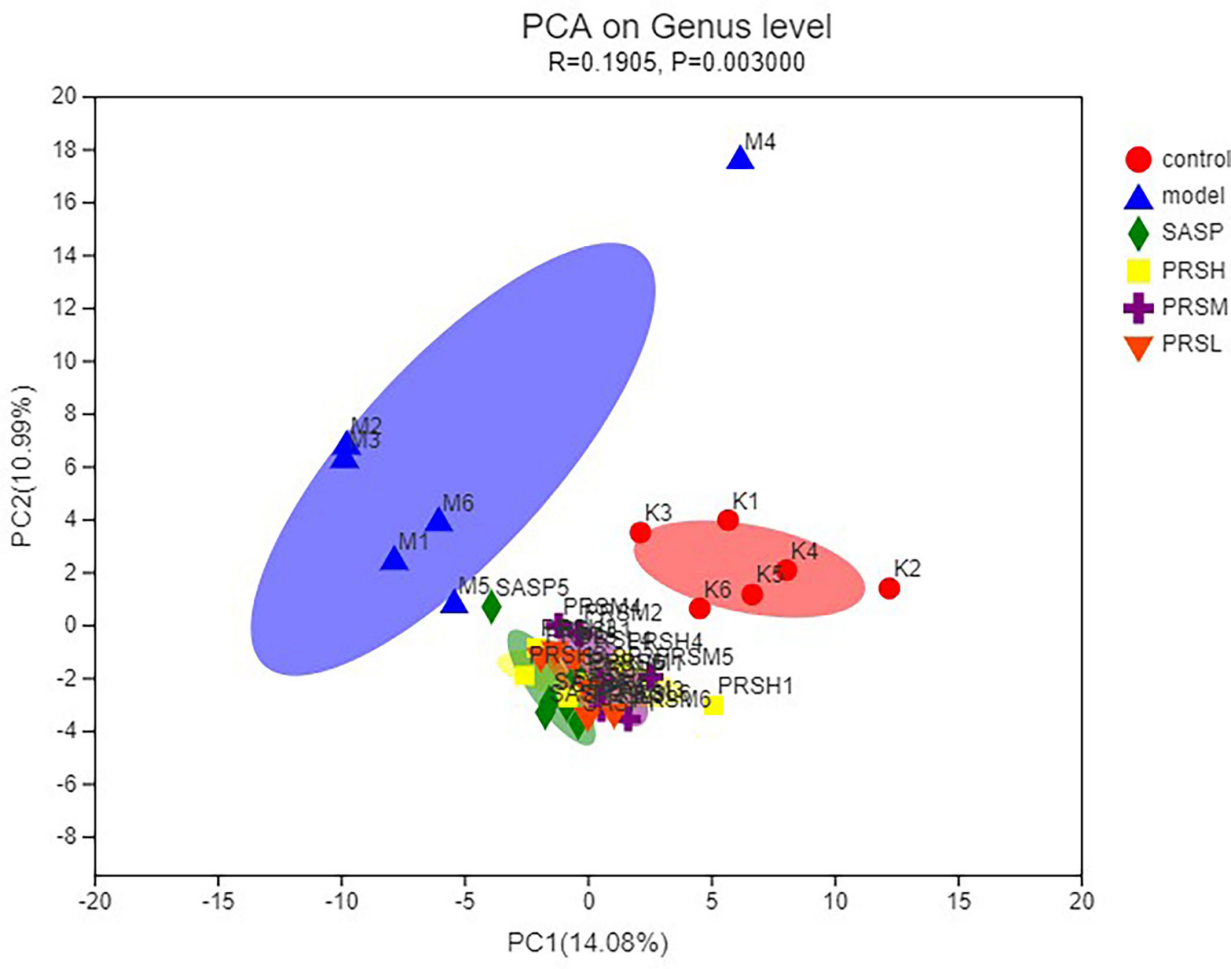
PCoA analysis of intestinal microbes in UC rats.

In this study, the OTU Venn diagram was used to analyze the OTU number and the cross of OTU species among groups. Principal component analysis (PCA) was used to detect the overall differences in bacterial abundance among the samples. The total specific OTUs in each group were as follows: 42/525 in the control group, 5/379 in the model group, 3/404 in the SASP group, 3/452 in the PRSH group, 4/455 in the PRSM group, and 4/462 in the PRSL group. This indicates that compared with the control group, the species richness of intestinal flora in the model group decreased; compared with the model group, the species richness of intestinal flora in the SASP group, PRSH group, PRSM group, and the PRSL groups increased. The results of PCA analysis of OTU abundance in each group showed that the control group and model group were significantly different from the SASP group, PRSH, PRSM, and PRSL, which indicated that compared with the model group, SASP group, and PRSH, PRSM, and PRSL groups gathered downward and tended to return to the control group, which indicated that SASP and PRS could promote the recovery of intestinal flora abundance and structure. There was a higher similarity between SASP and PRS, and the results are shown in **Figures 7**, **8**.

**Figure 9:**
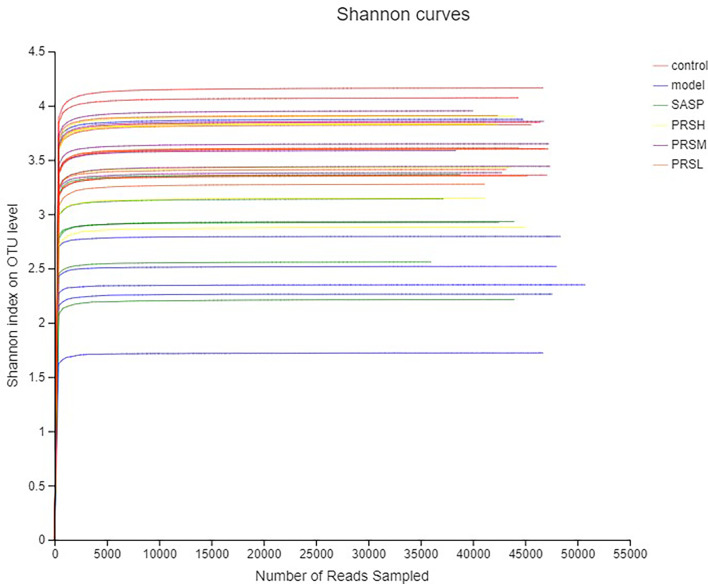
Shannon curves analysis of intestinal microbes in UC rats.

**Figure 10:**
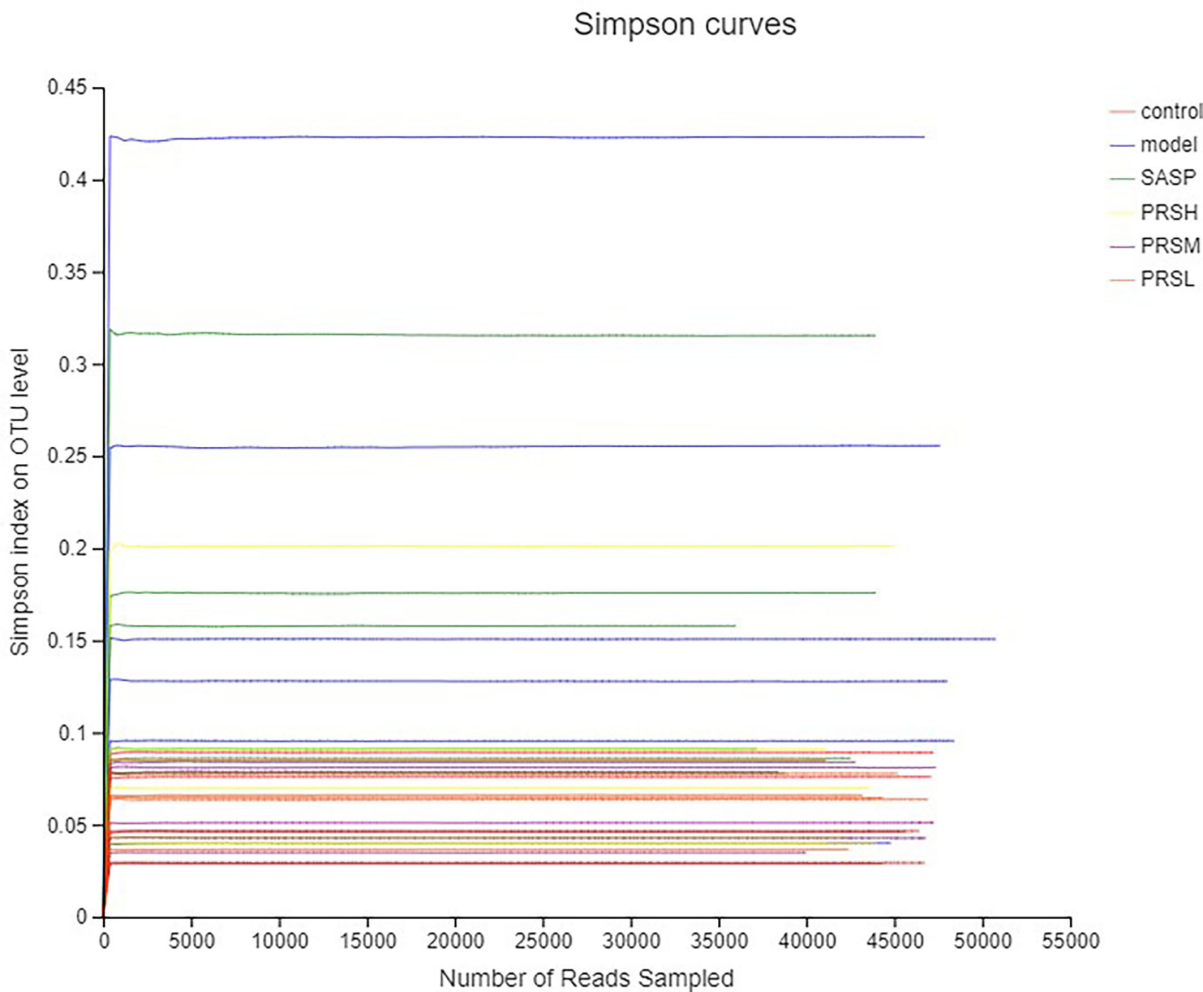
Simpson curves analysis of intestinal microbes in UC rats.

Alpha diversity is the analysis of species diversity in a single sample, including ACE, Chao1, Shannon, and Simpson indexes. These first three larger indexes and the last smaller one indicates that the species in the samples are more abundant. Ace and Chao1 index reflect the community richness of species in a single sample, while Shannon and Simpson index represents microbial diversity. The corresponding dilution curve is made by calculating the alpha diversity value of each group of samples, and the results of ACE, Chao1, and Shannon index showed that compared with the model group, ACE, Chao1, Shannon in the control group, SASP group, PRSH group, PRSM group, and PRSL group group were significantly higher than those in the model group, and there were no significant differences among the four groups (P > 0.05); Simpson index results: compared with the model group, the Simpson index of the control group, SASP group, PRSH group, PRSM group, and PRSL group were significantly lower than those in the model group (P < 0.05, P < 0.01). The above results showed that compared with the model group, the diversity of intestinal flora in SASP group, PRSH group, PRSM group, and PRSL group group increased, and the diversity of intestinal flora progressed to the control group. The results are shown in **Figures 9**, **10**.

**Figure 11:**
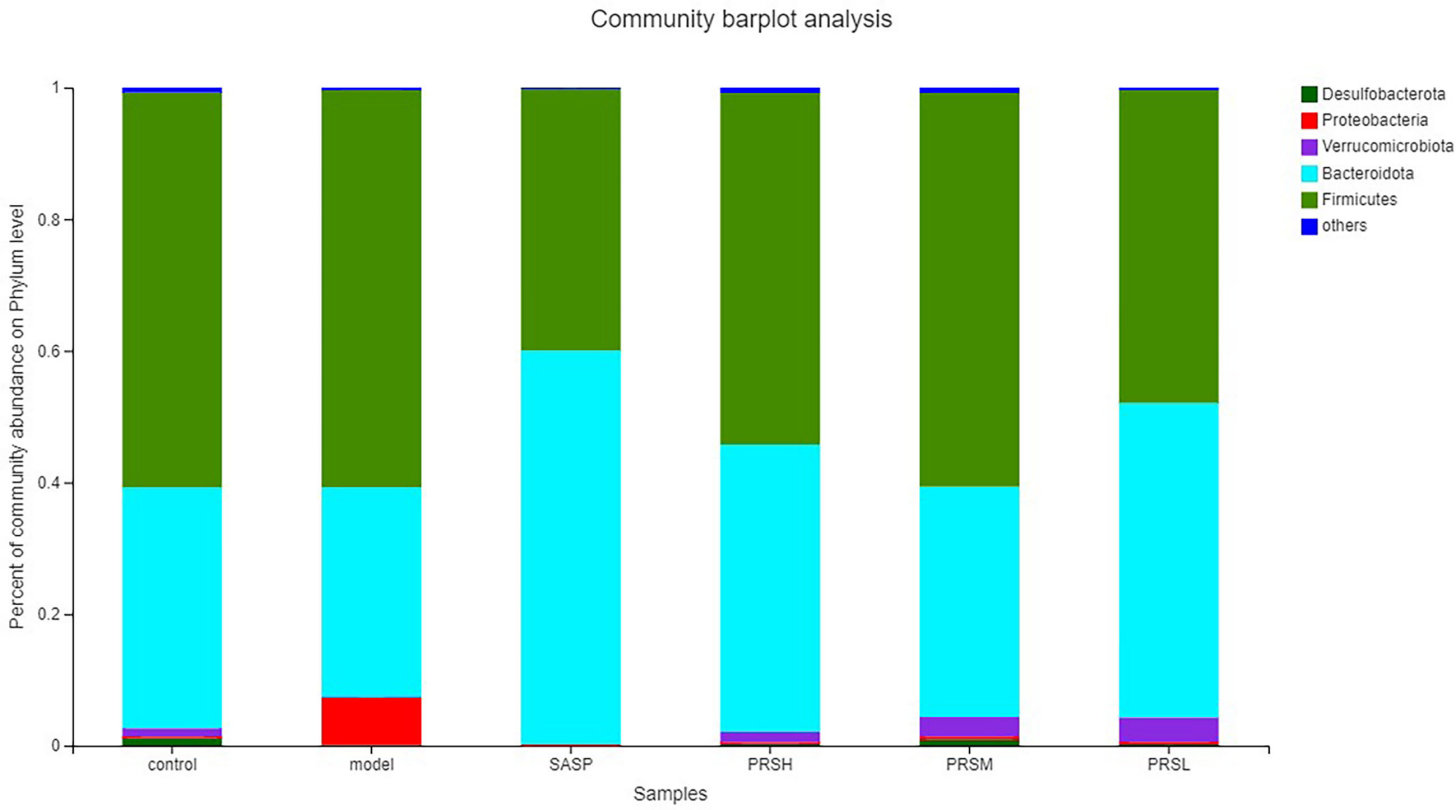
Community barplot analysis on phylum level.

**Figure 12:**
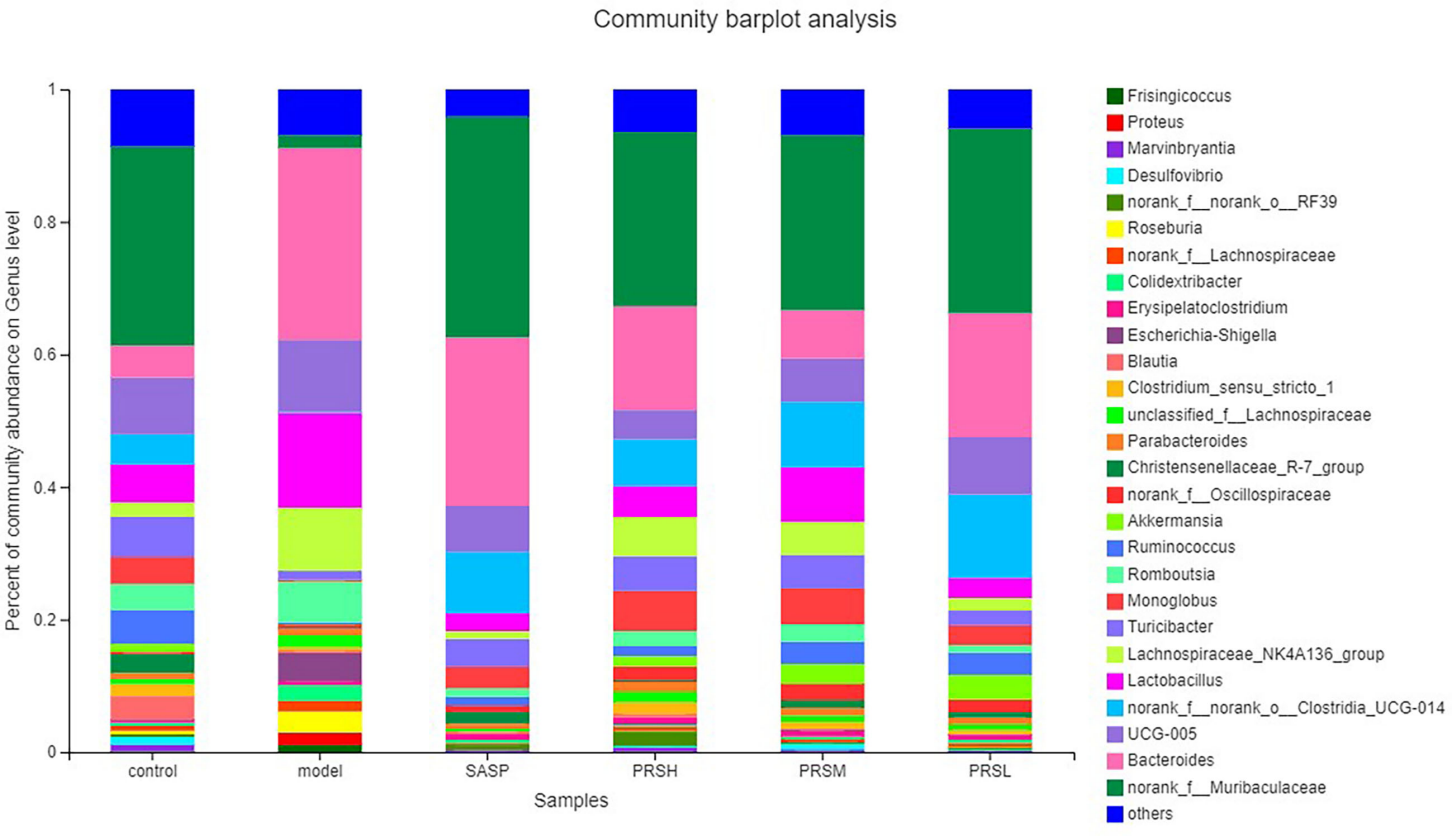
Community barplot analysis on genus level.

*Pulsatilla chinensis* (Bunge) Regel can treat schistosomiasis, amoebiasis, and inflammatory bacterial infections, and its biologically active ingredients are PRS, belonging to the pentacyclic triterpenes, oleanane type (Sparg et al., 2004; Zheng et al., 2010; Ip et al., 2015; Kang et al., 2018). The results of species analysis at the phylum classification level showed that Six phyla were detected at the phyla classification level, among which Firmicutes and Bacteroidetes accounted for the largest proportion; compared with the control group, the abundance of Verrucomicrobiota, Bacteroidetes, and Desulfobacteria in the model group decreased, while the abundance of Proteobacteria increased; Compared with the model group, the abundance of Firmicutes and Proteobacteria in SASP group was significantly decreased, and the abundance of Bacteroidetes was increased. The abundance of Bacteroidetes and verrucomicrobiota increased, and the abundance of Proteobacteria was decreased in the SASP group compared with the model group. The results of species analysis at genus classification level showed that compared with the control group, the bacterial flora of the model group changed significantly, the number of bacteria increased significantly, mainly containing Bacteroides, Lactobacillus, lachnospiraceae_NK4A136_group, romboutsia; and the number of bacteria decreased significantly, mainly containing norank_F_Muribaculaceae, norank_F_norank_O_Clostridia_UCG-014, Turicibacter, Monoglobus; Compared with the control group, the bacterial flora of SASP group changed significantly, and the number of bacteria decreased significantly, mainly containing Lactobacillus, lachnospiraceae_NK4A136_group, romboutsia, and Escherichia-Shigella. Furthermore, compared with the model group, the PRSH group, PRSM group, and PRSL group changed significantly, and the number of bacteria decreased significantly, mainly containing such as Bacteroides, Lactobacillus, lachnospiraceae_NK4A136_group, and romboutsia, and the number of bacteria increased significantly, mainly containing norank_F_muribaculaceae increased, as shown in **Figures 11**, **12**.

## Discussion

UC is a chronic disease characterized by local tissue damage, imbalance in intestinal flora, inflammation in the colon. In addition, untreated UC poses a serious risk of colon cancer development. The conventional treatment of UC is quite expensive except for corticosteroids; therefore, herbal medicines are mostly preferred due to their lower costs and good efficacy. In the present study, the DSS- induced UC animal model was used to determine the effects of PRS in managing UC by comparing it with the positive control SASP treatment, which is an antimicrobial agent widely used for the management of inflammatory bowel diseases (IBD). The normal intestinal flora acts as a barrier to protect the intestinal tract, and the changes in the intestinal flora composition may affect the intestinal microecology, thus affecting the immune and metabolic functions, which can lead to a variety of autoimmune and intestinal diseases, including UC (Sánchez-Fidalgo et al., 2010). The present study showed that both PRS and SASP groups had significantly higher colonic length and weight than the model group, and both could significantly improve the colonic ulcer and significantly reduce the mesenteric blood flow in UC rats. These results suggest that PRS had a significant therapeutic effect on DSS-induced UC. The standard treatment methods for UC, including anti-inflammatory and immunosuppressive drugs, mainly aim at downregulation of inflammation as well as suppression of immune response. Unfortunately, these drugs are associated with undesirable side effects and complications. Therefore, new molecules with high efficacy and safety, preferably of natural origin, are needed in UC treatment (Ko and Auyeung, 2014). In view of the excellent performance of pharmacodynamics, PRS and its monomer compounds in colitis treatment have great development potential.

The inferior mesenteric artery originates from the abdominal aorta’s anterior wall and supplies descending colon, sigmoid colon, rectum, and most transverse colon (Shirahama et al., 2001). It is worth noting that the intestinal blood supply is affected by various factors, including myogenic and local metabolic factors, autonomic nervous system, and vasoactive substances. The hemodynamic changes of mesentery may be caused by various intestinal diseases. The blood flow volume in the model group was significantly higher than that in the control group, SASP group, PRSH group, PRSM group, and PRSL group. The hemorheology results showed that the PRSH and PRSM could significantly reduce mesenteric blood flow and improve inflammatory reaction.

Intestinal flora plays an important role in the occurrence and development of UC. The severity of intestinal flora imbalance directly affects the progress rate of inflammation (Coqueiro et al., 2019). The results (**Figures 11**, **12**) of high-throughput sequencing showed that the dominant flora in each group was Bacteroides and Firmicutes at the phylum level. The abundance of Proteobacteria in the model group was significantly higher than that in the other groups. The abundance of verruco microbiota in the PRSH, PRSM, and PRSL groups was close to that of the control group and higher than that of the SASP group. At the genus level, the dominant bacteria in each group were norank_F_Muribaculaceae and Bacteroides. Compared with the model group, the three-dose groups of PRS can significantly improve the number of norank_F_Muribaculaceae, norank_F_norank_O_Clostridia_UCG-014 and decrease the number of Bacteroides. The norank_F_Muribaculaceae has been shown to improve intestinal mucositis in mice (Wang et al., 2016); norank_F_norank_O_Clostridia_ UCG-014 can tolerate gastric acid entering the intestine, promote the growth of beneficial bacteria, inhibit the harmful growth of the intestinal tract, restore intestinal flora, improve immunity, and promote the absorption, digestion, and absorption of nutrients. Bacteroides are an important clinical pathogen, which has been found in most anaerobic infections, with a mortality rate of more than 19%. When bacteria stay in the intestinal tract, they maintain a complex and beneficial relationship with the host, but when they escape this environment, they can cause serious pathological changes, including bacteremia and abscess formation in multiple body parts, and this bacteria is also related to the formation of UC (Wexler, 2007; Cui et al., 2018; Zhao et al., 2019). PRSH group and PRSM group can significantly increase the length of the colon and reduce colon weight. The results of the pathological sections of the colon showed that the three-dose groups of PRS could significantly improve the structure of colonic mucosa without obvious ulcer; PRS can improve DSS-induced UC and reduce the inflammatory response in a dose-dependent manner by reducing the abundance of Proteobacteria and increasing the abundance of Verrucomicrobiota. Our results suggest that PRS regulate intestinal flora, such as a significantly increasing beneficial bacteria norank_F_Muribaculaceae and norank_ F_norank_O_Clostridia_UCG-014 and decreasing the harmful bacteria Bacteroides, which may be the possible pathway for the excellent anti-UC effects of PRS.

## Conclusion

In conclusion, our study shows that PRS can significantly improve DSS-induced UC and reduce inflammation by regulating the composition and biodiversity of intestinal flora. The potential mechanism for the anti-UC activity of PRS may be related to the significant increase in beneficial bacteria norank_F_Muribaculaceae and norank_F_norank_O_Clostridia_UCG-014 and decrease in the harmful bacteria Bacteroides. The results showed that PRS and their monomer compounds had great potential in the treatment of UC and provides a certain basis for futuristic clinical studies on microboitic manipulation as a therapeutic approach for the treatment of various other intestinal diseases. In view of the excellent therapeutic effect of PRS on UC, we will consider PRS saponins as a possible prebiotic for UC treatment.

## Data Availability Statement

The datasets presented in this study can be found in online repositories. The names of the repository/repositories and accession number(s) can be found below: https://www.ncbi.nlm.nih.gov/bioproject/PRJNA755278.

## Ethics Statement

Animal care was as per the Guidelines for Animal Experiments of Jiangxi University of Chinese Medicine (Nanchang), and the experimental protocol was approved by the University’s Animal Ethics Committee.

## Author Contributions

YS, YL, and MZ designed, performed experiments, and wrote the manuscript. DS and ZA provided experimental funds. CJ and MY helped with several experimental procedures. JC and MG analyzed the metabolomics data. All authors contributed to the article and approved the submitted version.

## Funding

This research was financially supported by the National Natural Science Foundation of China (No.82074014, No.81860702), Jiangxi Province Nature Scientific Project (No. 20202BABL206142, 20202BAB206079), Science and Technology Key Project of Jiangxi Provincial Department of Education (GJJ201206, 181579), Science and technology project of Jiangxi Administration of Traditional Chinese Medicine (2020Z003), Innovation and entrepreneurship training program for College Students (202010412204), Jiangxi University of Traditional Chinese Medicine Graduate innovation Fund, (JZYC20S01), Foundation for Doctoral Research Initiation of Jiangxi University of Traditional Chinese Medicine (2018WBZR014).

## Conflict of Interest

The authors declare that the research was conducted in the absence of any commercial or financial relationships that could be construed as a potential conflict of interest.

## Publisher’s Note

All claims expressed in this article are solely those of the authors and do not necessarily represent those of their affiliated organizations, or those of the publisher, the editors and the reviewers. Any product that may be evaluated in this article, or claim that may be made by its manufacturer, is not guaranteed or endorsed by the publisher.

## Abbreviations

PRS, *Pulsatilla chinensis* saponins; DSS, dextran sulfate sodium; UC, ulcerative colitis; TCM, traditional Chinese medicine; SASP, Salicylazosulfapyrid; SD, Sprague-Dawley; PRSH, Pulsatilla chinensis saponins high dose group; PRSM, Pulsatilla chinensis saponins middle dose group; PRSL, Pulsatilla chinensis saponins low dose group; CMC-Na, Carboxymethylcellulose sodium; OTU, Operational taxonomic units; PCoA, principal coordinate analysis; HE Staining, hematoxylin staining.

## References

[B25] Bloemendaal F. M. Becker M. Koelink P. J. Van D. Bemelman W. A. D’Haens G. R. A. M.. (2020). Dop82 Macrophages in Crohn’s Disease Mesentery Are Predominantly Inflammatory and Produce Calprotectin. J. Crohns Colitis. 14, 121–122. doi: 10.1093/ecco-jcc/jjz203.121

[B17] Borody T. Warren E. Leis S. Surace R. Ashman O. (2003). Treatment of Ulcerative Colitis Using Fecal Bacteriotherapy. J. Clin. Gastroenterol. 37 (1), 42–47. doi: 10.1097/00004836-200307000-00012 12811208

[B33] Coqueiro A. Y. Raizel R. Bonvini A. Tirapegui J. Rogero M. (2019). Probiotics for Inflammatory Bowel Diseases: A Promising Adjuvant Treatment. Int. J. Food Sci. 70 (1), 20–29. doi: 10.1080/09637486.2018.1477123 29804478

[B37] Cui H. Cai Y. Wang L. Jia B. Li J. Zhao S.. (2018). Berberine Regulates Treg/Th17 Balance to Treat Ulcerative Colitis Through Modulating the Gut Microbiota in the Colon. Front. Pharmacol. 9, 571. doi: 10.3389/fphar.2018.00571 29904348 PMC5991375

[B13] Feng W. Ao H. Peng C. Yan D. (2019). Gut Microbiota, A New Frontier to Understand Traditional Chinese Medicines. Pharmacol. Res. 142, 176–191. doi: 10.1016/j.phrs.2019.02.024 30818043

[B20] Frank D. N. Pace N. R. (2008). Gastrointestinal Microbiology Enters the Metagenomics Era. Curr. Opin. Gastroenterol. 24 (1), 4–10. doi: 10.1124/mol.106.032656 18043225

[B6] Guisen L. (2015). Study on Pulsatilla Decoction in Treatment of Ulcerative Colitis. China Contin. Med. Educ. 7, 249–250. doi: CNKI:SUN:JXUY.0.2015-10-221

[B19] Himmel M. E. Yao Y. Orban P. C. Steiner T. S. Levings M. K. (2012). Regulatory T-Cell Therapy for Inflammatory Bowel Disease: More Questions Than Answers. Immunology 136 (2), 115–122. doi: 10.1111/j.1365-2567.2012.03572.x 22348589 PMC3403270

[B15] Huang G. Ye L. Du G. Huang Y. Wu Y. Ge S. (2017). Effects of Curcumin Plus Soy Oligosaccharides on Intestinal Flora of Rats With Ulcerative Colitis. Cell. Mol. Biol. (Noisy-le-grand) 63 (7), 20–25. doi: 10.14715/cmb/2017.63.7.3 28838334

[B28] Ip F. C. Fu W. Y. Cheng E. Y. Tong E. P. Lok K. C. Liang Y.. (2015). Anemoside A3 Enhances Cognition Through the Regulation of Synaptic Function and Neuroprotection. Neuropsychopharmacolo 40 (8), 1877–1887. doi: 10.1038/npp.2015.37 PMC4839511 25649278

[B14] Jiang L. Lv J. Liu J. Hao X. Ren F. Guo H.. (2018). Donkey Milk Lysozyme Ameliorates Dextran Sulfate Sodium-Induced Colitis by Improving Intestinal Barrier Function and Gut Microbiota Composition. J. Funct. Foods 48, 144–152. doi: 10.1016/j.jff.2018.07.005

[B26] Kang N. Shen W. Gao H. Feng Y. Zhu W. Yang S.. (2018). Antischistosomal Properties of Hederacolchiside A1 Isolated From Pulsatilla Chinensis. Molecules 23 (6), 1431. doi: 10.3390/molecules23061431 PMC6099544 29899232

[B31] Ko K. J. Auyeung K. K. (2014). Inflammatory Bowel Disease: Etiology, Pathogenesis and Current Therapy. Curr. Pharm. Des. 20 (7), 1082–1096. doi: 10.2174/13816128113199990416 23782147

[B4] Liu Q. Chen W. Jiao Y. Hou J. Wu Q. Liu Y.. (2014). *Pulsatilla Saponin* A, an Active Molecule From Pulsatilla Chinensis, Induces Cancer Cell Death and Inhibits Tumor Growth in Mouse Xenograft Models. J. Surg. Res. 188, 387–395. doi: 10.1016/j.jss.2014.01.026 24576780

[B22] Liu Y. Song Y. Xu Q. Su D. Feng Y. Li X.. (2013). Validated Rapid Resolution LC-ESI-MS/MS Method for Simultaneous Determination of Five Pulchinenosides From Pulsatilla Chinensis (Bunge) Regel in Rat Plasma: Application to Pharmacokinetics and Bioavailability Studies. J. Chromatogr. B. Analyt. Technol. BioMed. Life Sci. 942-943, 141–150. doi: 10.1016/j.jchromb.2013.10.036 24269908

[B16] Oshitani N. Hato F. Kitagawa S. Maeda K. Higuchi K. Matsumoto T. (2003). Analysis of Intestinal HLA-DR Bound Peptides and Dysregulated Immune Responses to Enteric Flora in the Pathogenesis of Inflammatory Bowel Disease. Int. J. Mol. Med. 11 (1), 99–104. doi: 10.3892/ijmm.11.1.99 12469227

[B30] Sánchez-Fidalgo S. Cárdeno A. Villegas I. Talero E. de la Lastra C. A. (2010). Dietary Supplementation of Resveratrol Attenuates Chronic Colonic Inflammation in Mice. Eur. J. Pharmacol. 633 (1-3), 78–84. doi: 10.1016/j.ejphar.2010.01.025 20132809

[B21] Shan B. Ai Z. Zeng S. Song Y. Song J. Zeng Q.. (2020). Gut Microbiome-Derived Lactate Promotes to Anxiety-Like Behaviors Through GPR81 Receptor-Mediated Lipid Metabolism Pathway. Psychoneuroendocrinology 117, 104699. doi: 10.21203/rs.2.17202/v1 32402927

[B11] Shen Z. Zhu C. Quan Y. Yang Z. Wu S. Luo W.. (2018). Relationship Between Intestinal Microbiota and Ulcerative Colitis: Mechanisms and Clinical Application of Probiotics and Fecal Microbiota Transplantation. World J. Gastroenterol. 24, 5–14. doi: 10.3748/wjg.v24.i1.5 29358877 PMC5757125

[B100] Shirahama M. Ishibashi H. Onohara S. Miyamoto Y. (2001). Application of Color Doppler Ultrasonography to Ulcerative Colitis. J. Med. Ultrason. 30 (1), 39–44. doi: 10.1007/BF02485168 27285153

[B23] Song Y. Shan B. Li H. Feng B. Peng H. Jin C.. (2019). Safety 615 Investigation of Pulsatilla Chinensis Saponins From Chronic Metabonomic Study of Serum Biomedical 616 Changes in Oral Treated Rat. J. Ethnopharmacol. 235, 435–445. doi: 10.1016/j.phymed.2020.153265 30703498

[B8] Song Y. Shan B. Zeng S. Zhang J. Jin C. Liao Z.. (2021). Raw and Wine Processed Schisandra Chinensis Attenuate Anxiety Like Behavior via Modulating Gut Microbiota and Lipid Metabolism Pathway. J. Ethnopharmacol. 266:113426. doi: 10.1016/j.jep.2020.113426 33007392

[B29] Sparg S. G. Light M. E. van Staden J. (2004). Biological Activities and Distribution of Plant Saponins. J. Ethnopharmacol. 94 (2-3), 219–243. doi: 10.1016/j.jep.2004.05.016 15325725

[B1] State Pharmacopoeia Commission. (2020). Chinese Pharmacopoeia (Beijing: China Med. Sci. Technol. Press), 1088.

[B9] Su D. Liao Z. Feng B. Wang T. Shan B. Zeng Q.. (2020). *Pulsatilla Chinensis* Saponins Cause Liver Injury Through Interfering Ceramide/Sphingomyelin Balance That Promotes Lipid Metabolism Dysregulation and Apoptosis. Phytomedicine 76, 153265. doi: 10.1016/j.phymed.2020.153265 32575028

[B3] Tong X. Han L. Duan H. Cui Y. Feng Y. Zhu Y.. (2017). The Derivatives of Pulsatilla Saponin A, a Bioactive Compound From *Pulsatilla* Chinensis: Their Synthesis, Cytotoxicity, Haemolytic Toxicity and Mechanism of Action. Eur. J. Med. Chem. 129, 325–336. doi: 10.1016/j.ejmech.2017.02.025 28237662

[B10] Ungaro R. Mehandru S. Allen P. B. Peyrin-Biroulet L. Colombel J. (2016). Ulcerative Colitis. Lancet 389, 1756–1770. doi: 10.1016/S0140-6736(16)32126-2 27914657 PMC6487890

[B18] Venturi A. Gionchetti P. Rizzello F. Johansson R. Zucconi E. Brigidi P. (1999). Impact on the Composition of the Faecal Flora by a New Probiotic Preparation: Preliminary Data on Maintenance Treatment of Patients With Ulcerative Colitis. Aliment. Pharmacol. Ther. 13 (8), 1103–1108. doi: 10.1046/j.1365-2036.1999.00560.x 10468688

[B7] Wang X. Fan F. Cao Q. (2016). Modified Pulsatilla Decoction Attenuates Oxazolone-Induced Colitis in Mice Through Suppression of Inflammation and Epithelial Barrier Disruption. Mol. Med. Rep. 14 (2), 1173–1179. doi: 10.3892/mmr.2016.5358 27278299 PMC4940073

[B24] Wang D. Mei T. Yang G. Qiu J. Fu C. Shao S.. (2020). Study on Therapeutic Mechanism of Epimedium on Ulcerative Colitis in Rats. Chin. J. Integr. Tradit. Western Nephrol. 26, 817–822.

[B34] Wang L. Wang R. Wei G. Zhang R. Zhu Y. Wang Z.. (2020). Cryptotanshinone Alleviates Chemotherapy-Induced Colitis in Mice With Colon Cancer via Regulating Fecal-Bacteria-Related Lipid Metabolism. Pharmacol. Res. 163, 105232. doi: 10.1016/j.phrs.2020.105232 33027716

[B35] Wexler H. M. (2007). Bacteroides: The Good, the Bad, and the Nitty-Gritty. Clin. Microbiol. Rev. 20 (4), 593–621. doi: 10.1128/CMR.00008-07 17934076 PMC2176045

[B12] Wirtz S. Neurath M. F. (2017). Mouse Models of Inflammatory Bowel Disease. Adv. Drug Deliv. Rev. 59 (17), 1073–1083. doi: 10.1016/j.addr.2007.07.003 17825455

[B2] Xu Q. Chen Z. Ding X. Lu B. Ji C. Shu Z.. (2011). Three New Triterpenoids From Pulsatilla Chinensis (Bunge) Regel and Their Cytotoxic Activities. Heterocycles 83, 2365–2372. doi: 10.1002/chin.201204170

[B36] Zhao H. Cheng N. Zhou W. Chen S. Wang Q. Gao H. (2019). Honey Polyphenols Ameliorate DSS-Induced Ulcerative Colitis via Modulating Gut Microbiota in Rats. Mol. Nutr. Food Res. Dec. 63 (23), e1900638. doi: 10.1002/mnfr.201900638 31533201

[B27] Zheng Y. Zhou F. Wu X. Wen X. Li Y. Yan B.. (2010). 23-Hydroxybetulinic Acid From Pulsatilla Chinensis (Bunge) Regel Synergizes the Antitumor Activities of Doxorubicin *In Vitro* and *In Vivo* . J. Ethnopharmacol. 128 (3), 615–622. doi: 10.1016/j.jep.2010.02.004 20176097

