# Supplementary figures and images for: Pulsatilla chinensis Saponins Ameliorate Inflammation and DSS-Induced Ulcerative Colitis in Rats by Regulating the Composition and Diversity of Intestinal Flora
Source: Front Cell Infect Microbiol. 2021 Nov 5;11:728929. doi: 10.3389/fcimb.2021.728929 (PMC8602866; doi:10.3389/fcimb.2021.728929)

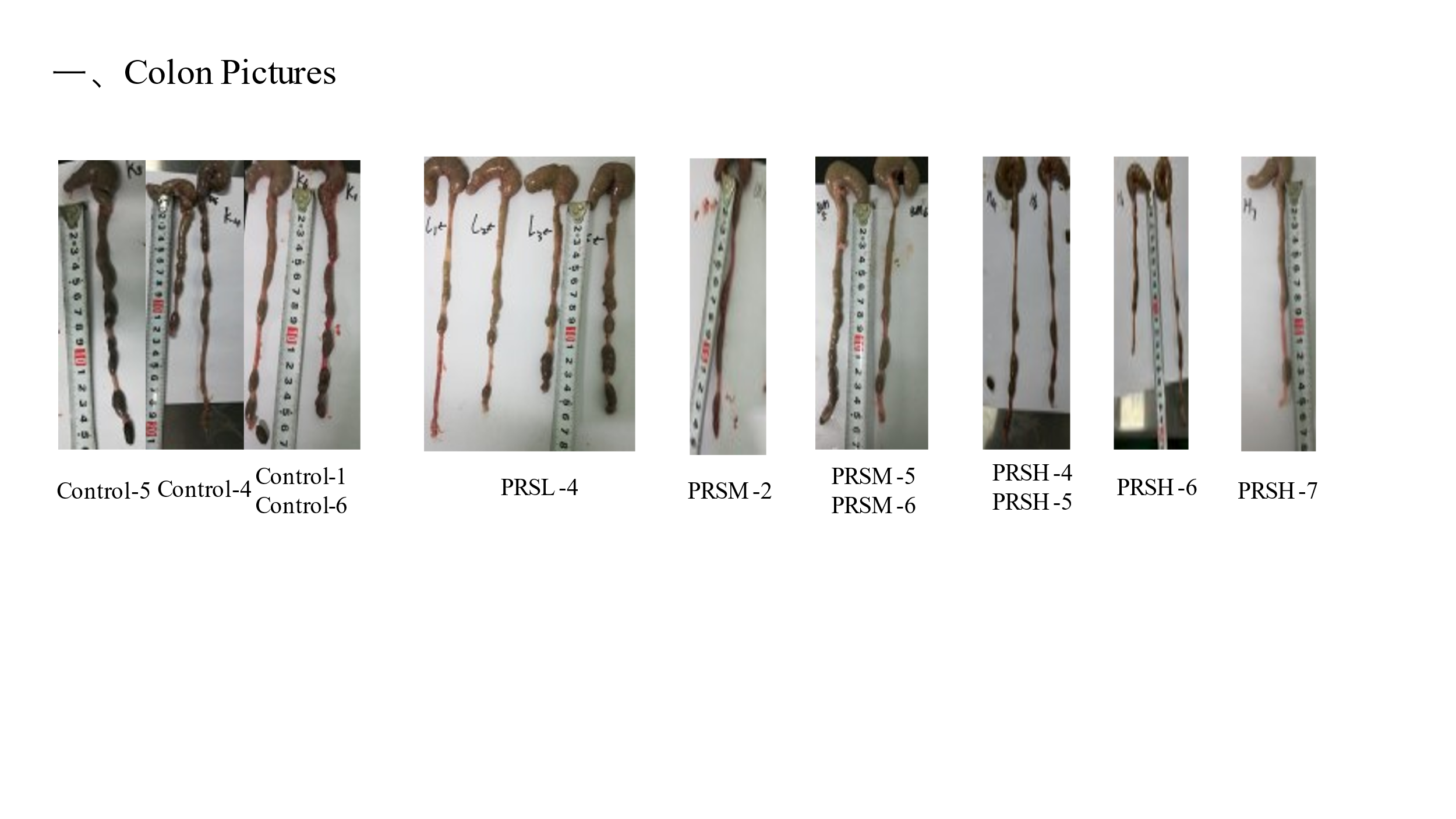


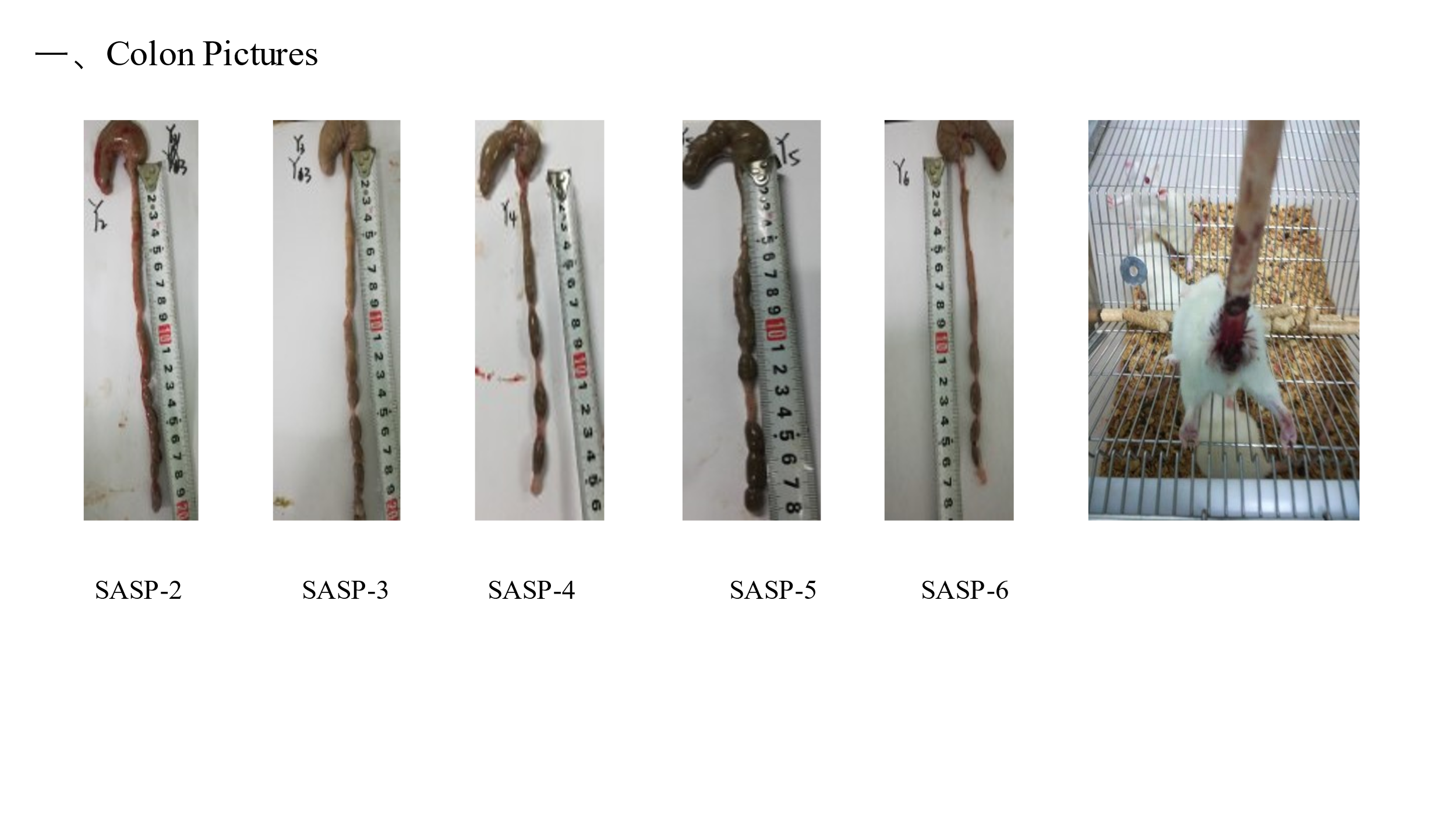


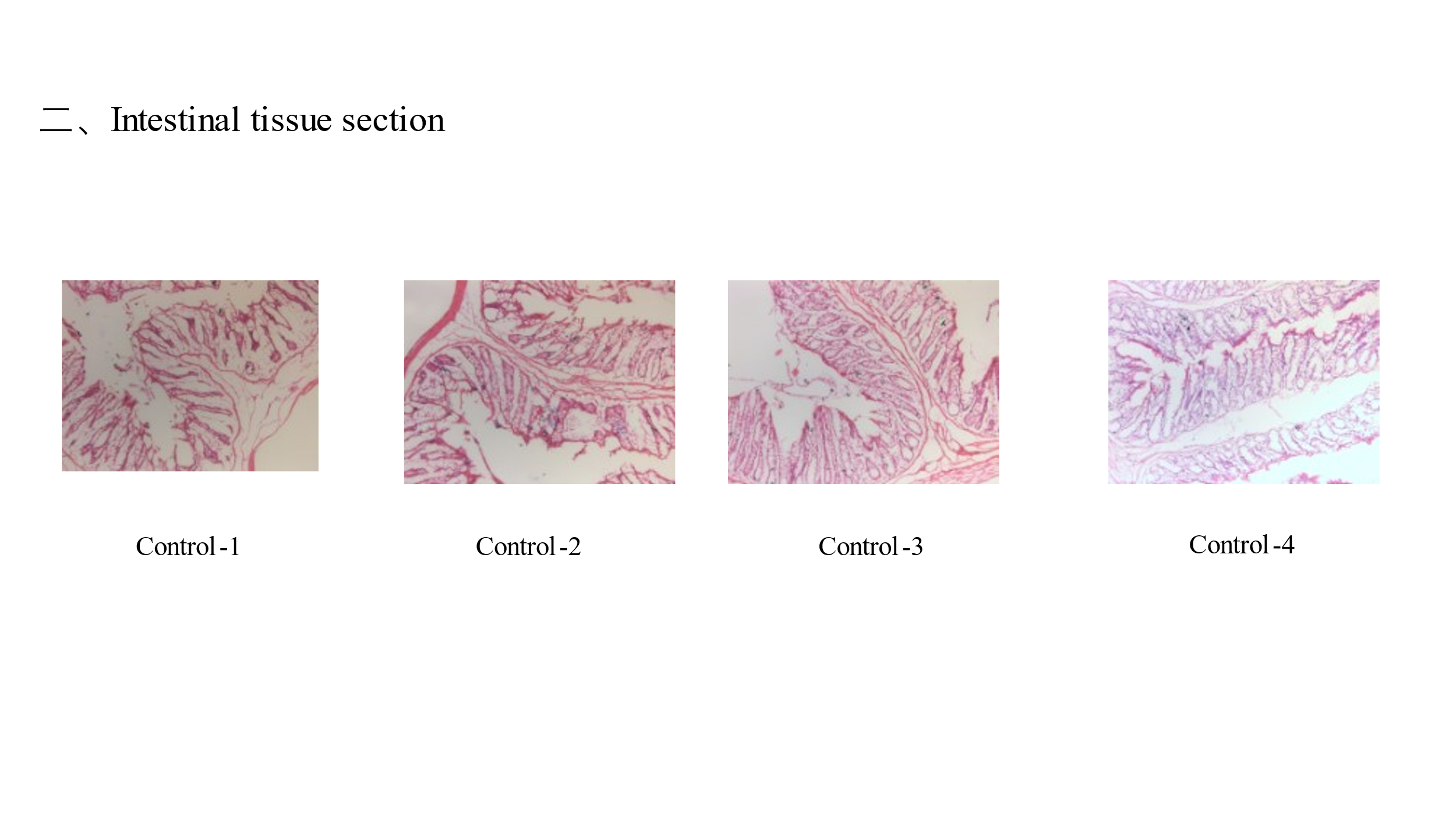


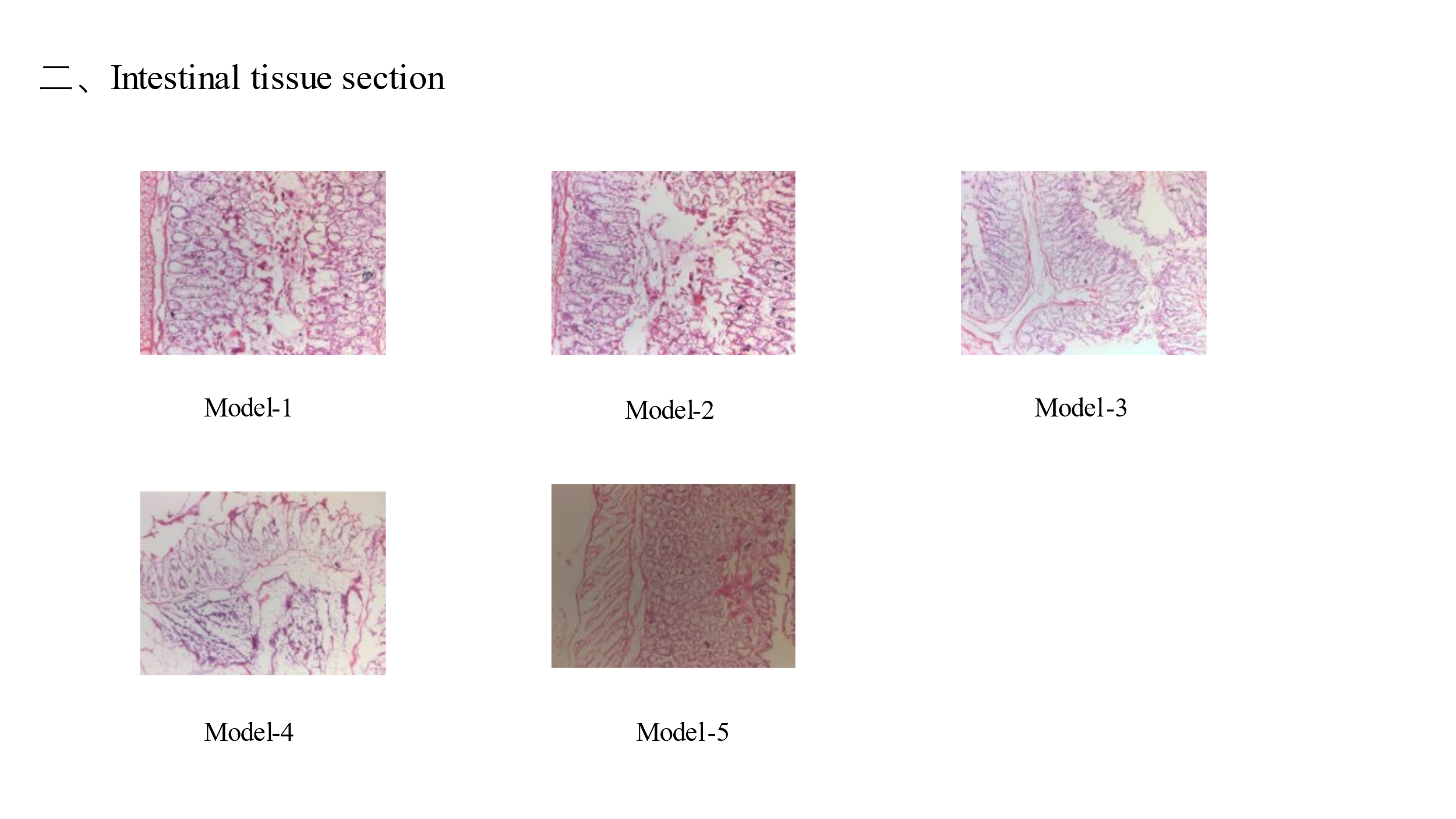


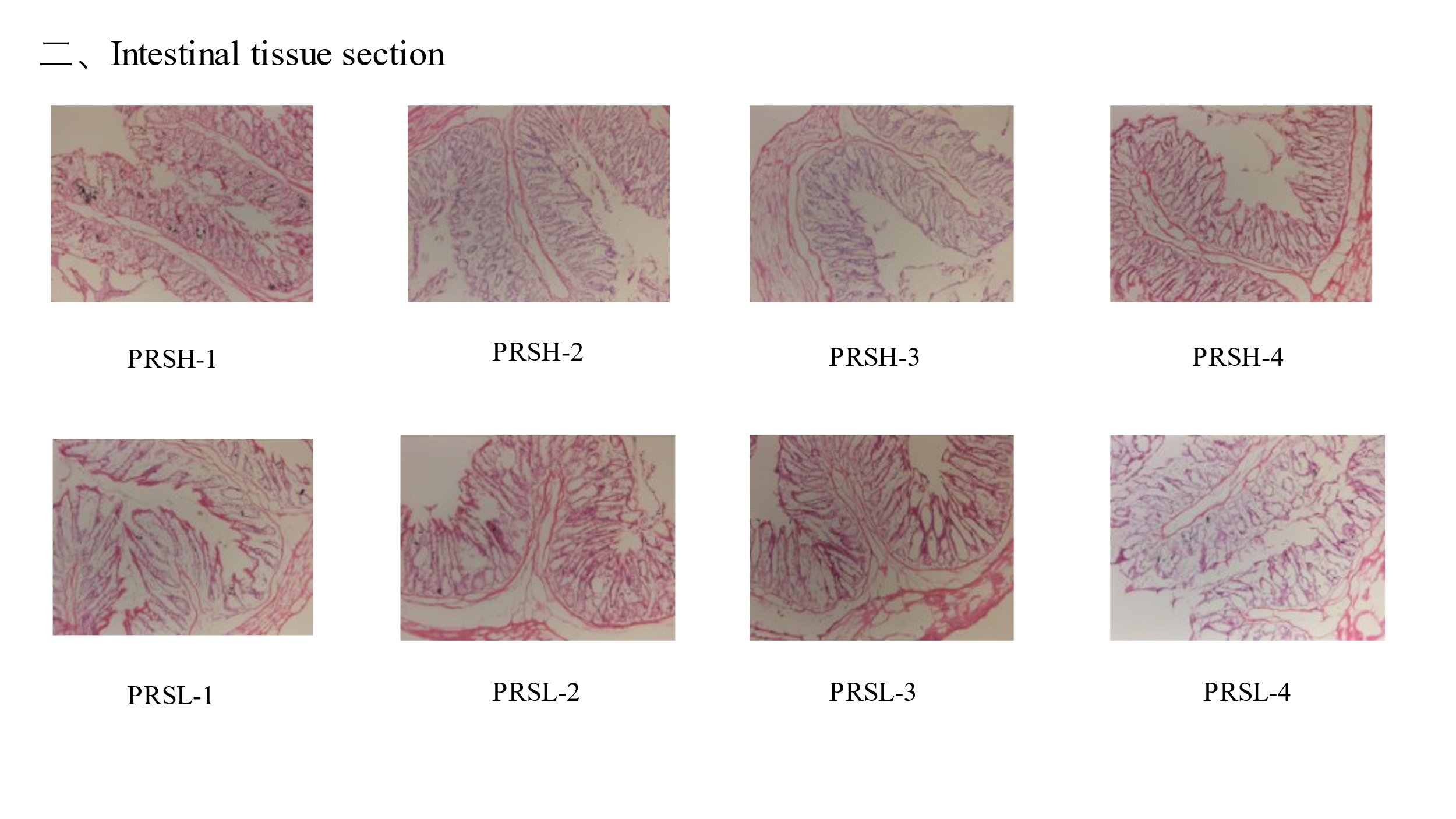


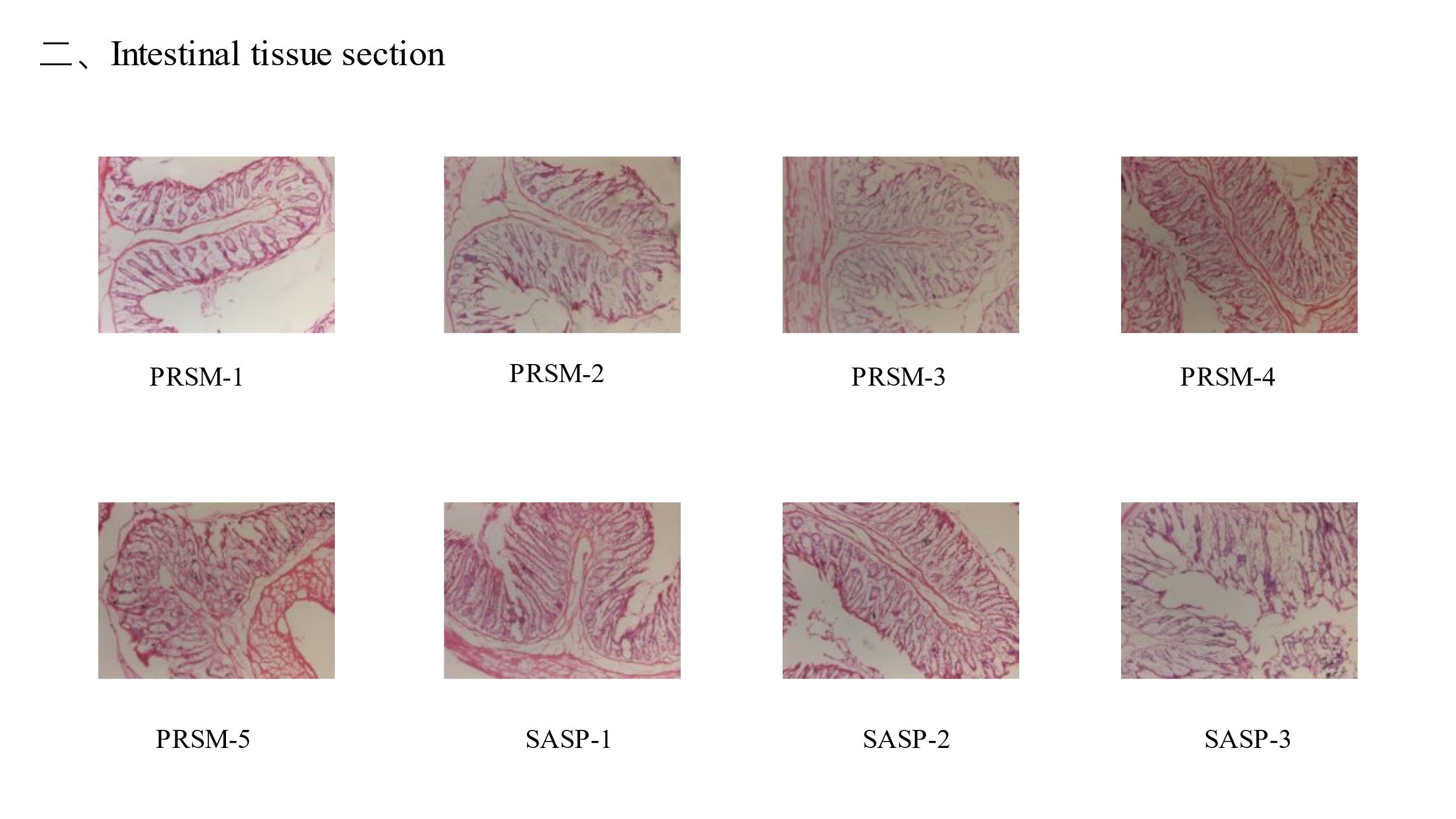


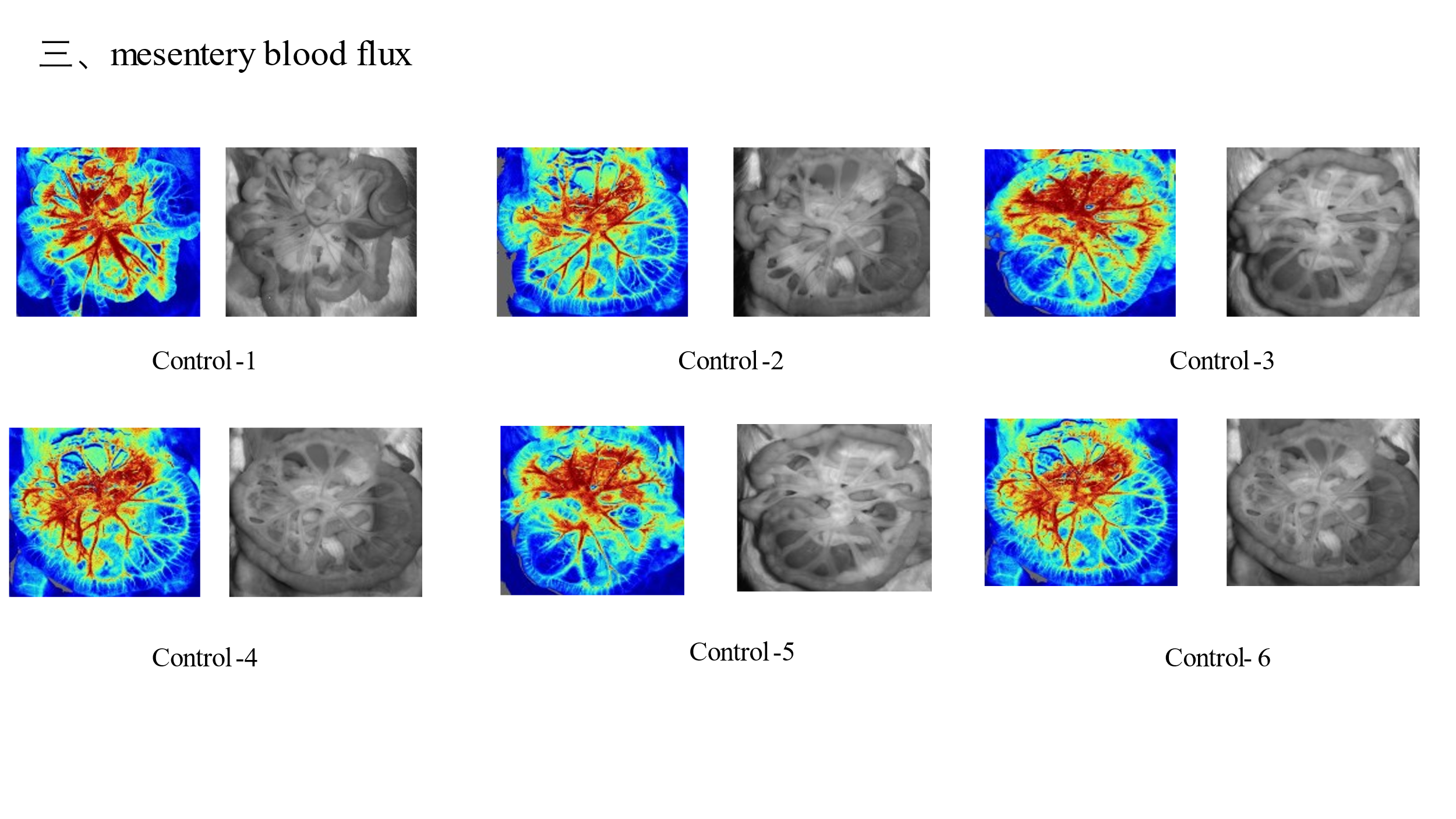


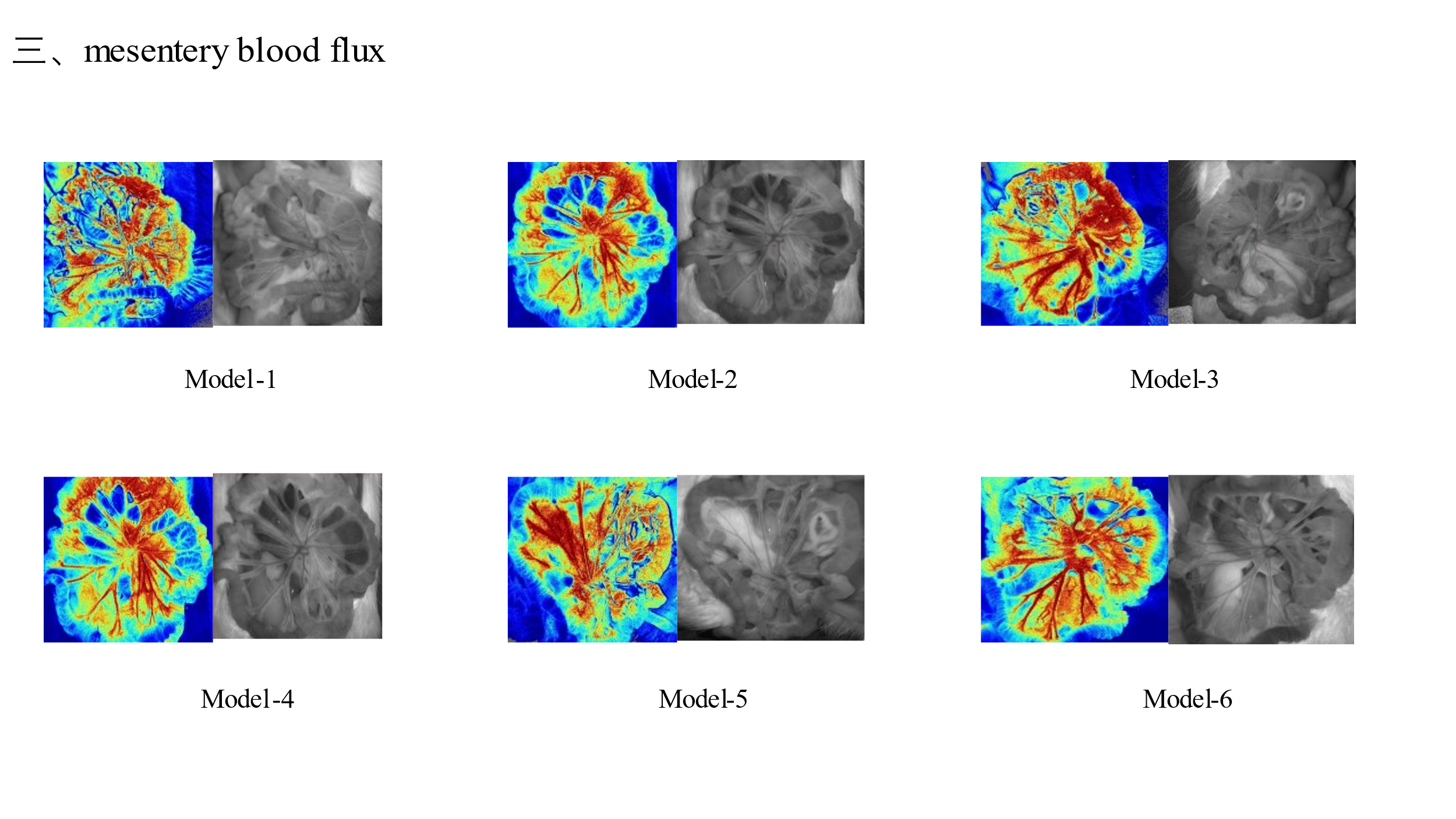


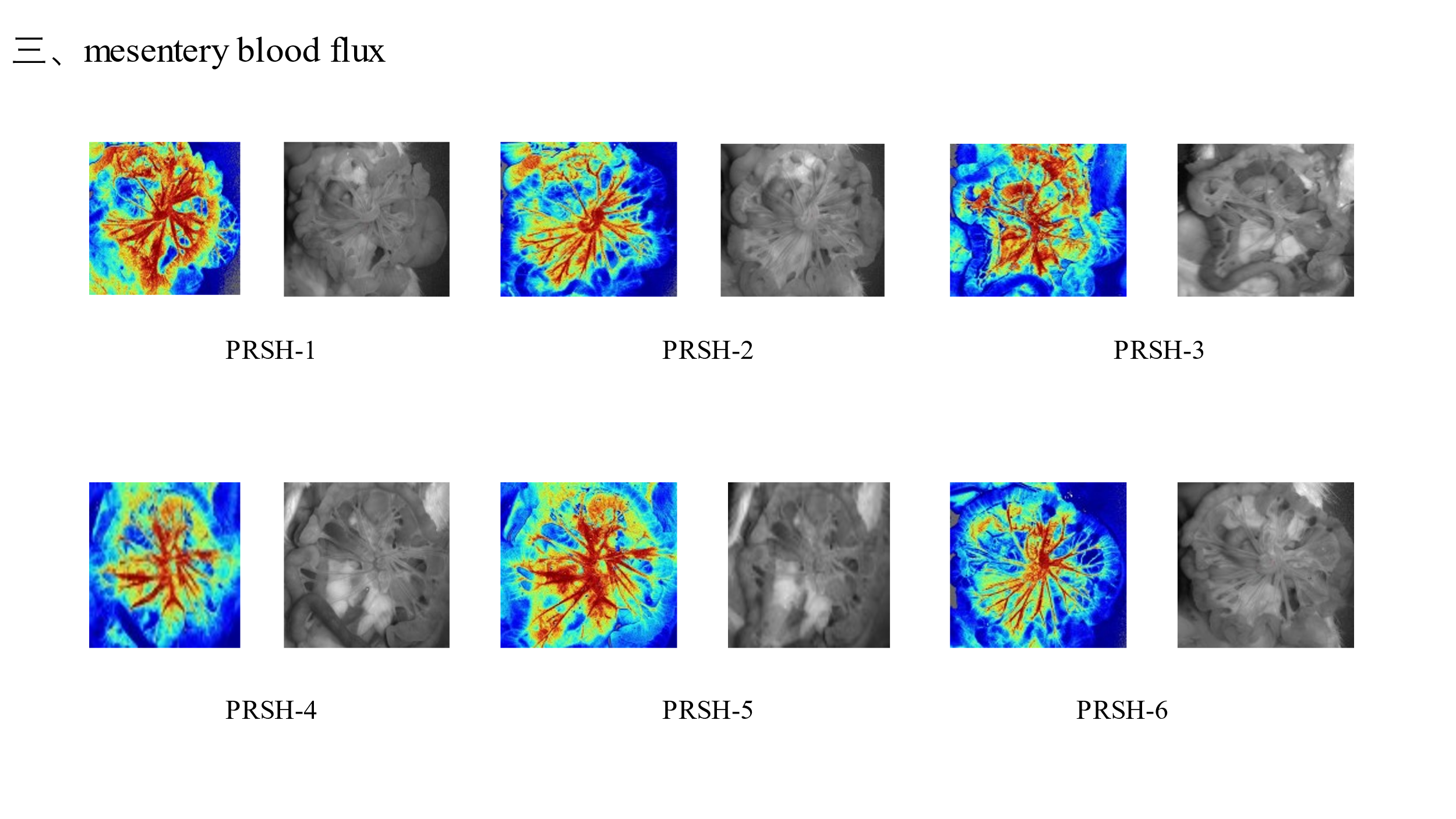


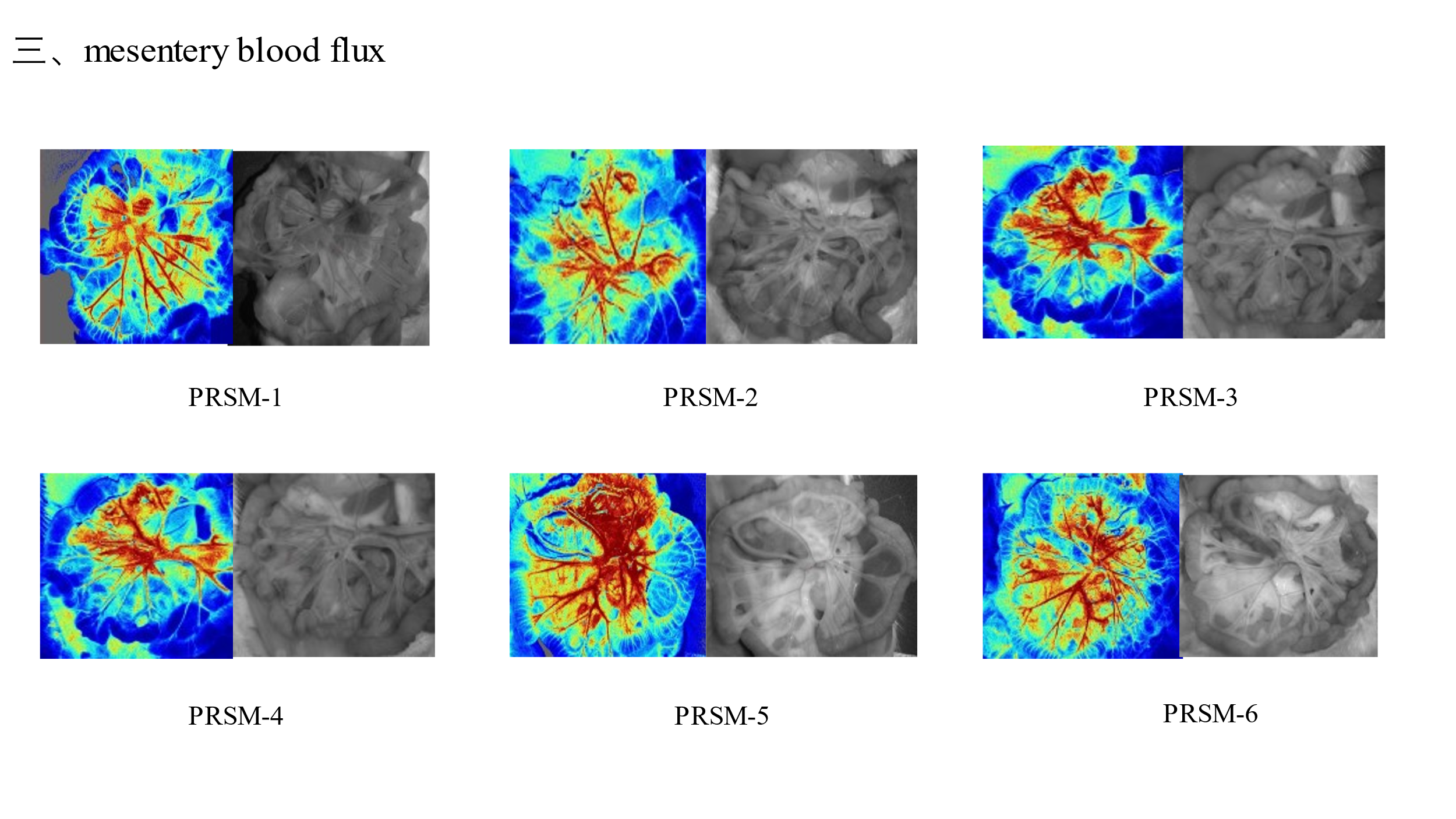


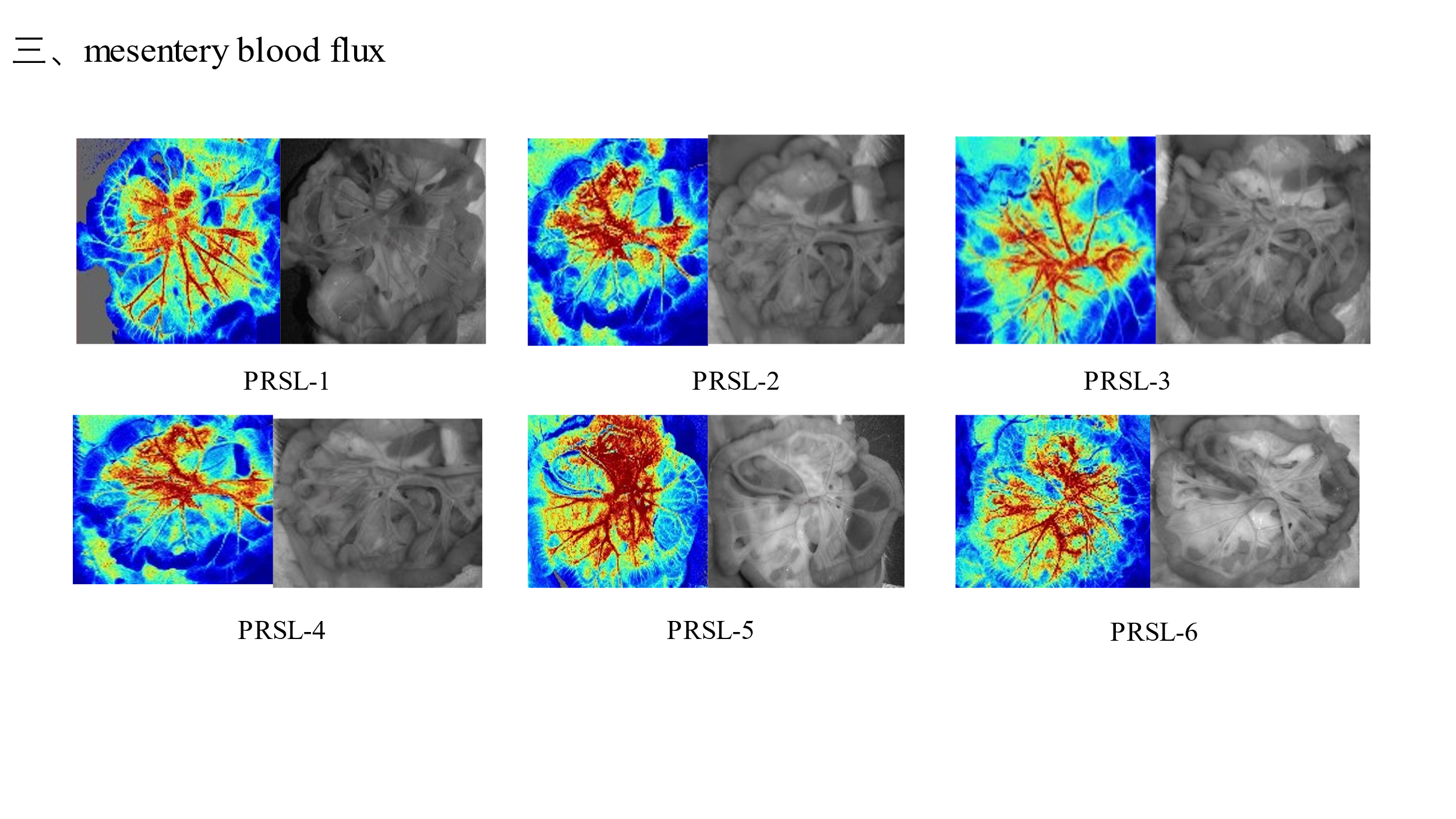


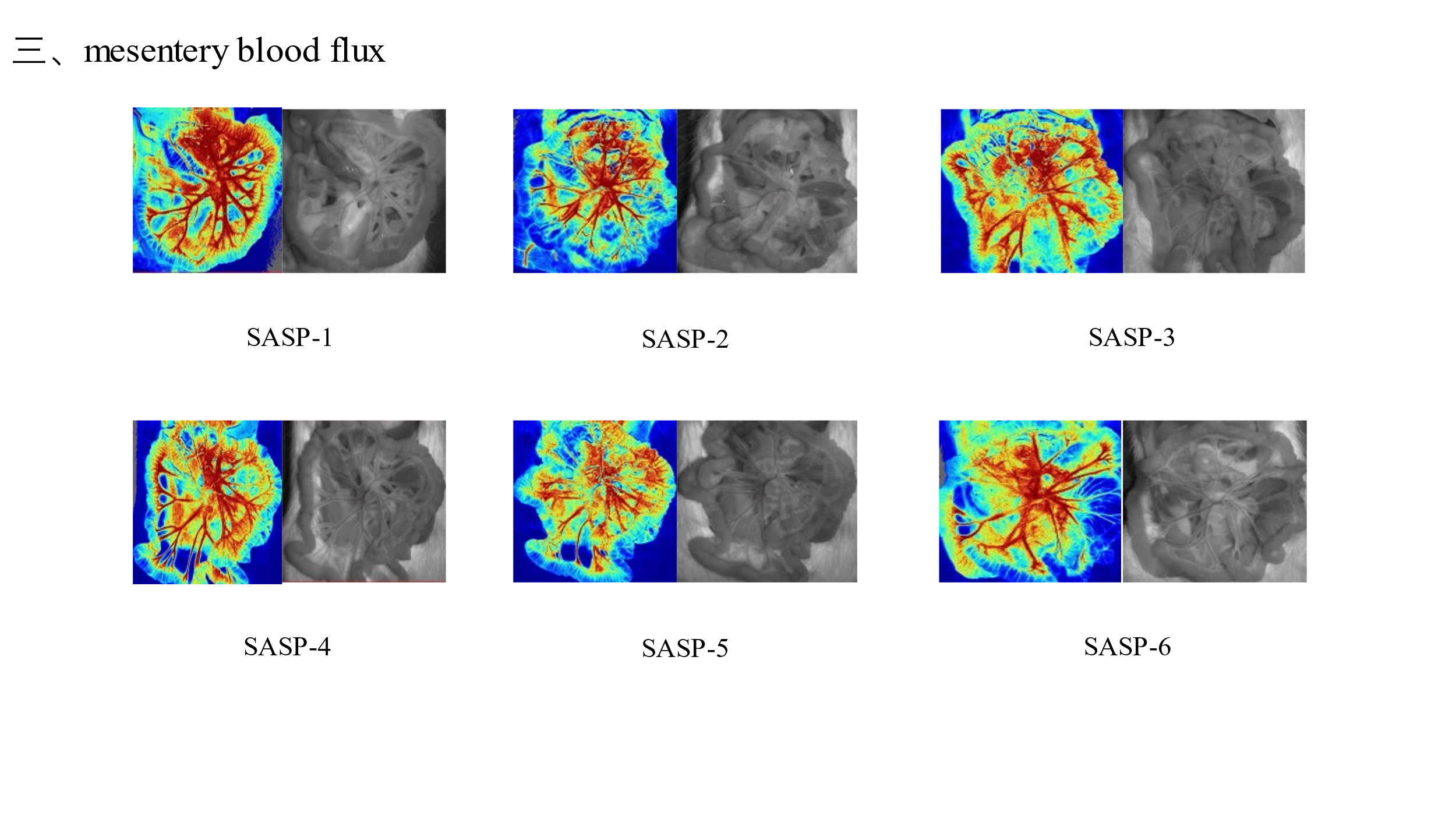

Supplement: Supplementary file 14 [file Table_14.docx]
